# The correlation theory of the chemical bond

**DOI:** 10.1038/s41598-017-02447-z

**Published:** 2017-05-22

**Authors:** Szilárd Szalay, Gergely Barcza, Tibor Szilvási, Libor Veis, Örs Legeza

**Affiliations:** 1 Strongly Correlated Systems “Lendület” Research Group, Institute for Solid State Physics and Optics, MTA Wigner Research Centre for Physics, H-1121 Budapest, Konkoly-Thege Miklós út 29-33, Hungary; 2 Department of Chemical and Biological Engineering, University of Wisconsin-Madison, 1415 Engineering Drive, Madison, Wisconsin 53706 United States; 3 Department of Inorganic and Analytical Chemistry, Budapest University of Technology and Economics, H-1111 Budapest, Szent Gellért tér 4, Hungary; 4 J. Heyrovský Institute of Physical Chemistry, Academy of Sciences of the Czech Republic, CZ-18223 Prague, Czech Republic

## Abstract

The quantum mechanical description of the chemical bond is generally given in terms of delocalized bonding orbitals, or, alternatively, in terms of correlations of occupations of localised orbitals. However, in the latter case, multiorbital correlations were treated only in terms of two-orbital correlations, although the structure of multiorbital correlations is far richer; and, in the case of bonds established by more than two electrons, multiorbital correlations represent a more natural point of view. Here, for the first time, we introduce the true multiorbital correlation theory, consisting of a framework for handling the structure of multiorbital correlations, a toolbox of true multiorbital correlation measures, and the formulation of the multiorbital correlation clustering, together with an algorithm for obtaining that. These make it possible to characterise quantitatively, how well a bonding picture describes the chemical system. As proof of concept, we apply the theory for the investigation of the bond structures of several molecules. We show that the non-existence of well-defined multiorbital correlation clustering provides a reason for debated bonding picture.

## Introduction

Since quantum theory is a probabilistic theory, it is not surprising that using concepts of quantum information theory^1, 2^ turns out to be fruitful in several fields of research in which quantum theory is involved. Maybe the most important notion in a probabilistic theory is correlation^3^, and, in quantum systems, also entanglement^4, 5^. Taking their investigation as a guiding principle has already led to important achievements in several fields of research^3, 6–8^, recently also in quantum chemistry^6–20^.

The notion of chemical bond^21^ is a very useful concept in chemistry. It originated at the beginning of chemistry, it is expressive for the classically thinking mind, and the errors arising from the approximative nature of the concept can often be ignored. In the first half of the twentieth century, however, we learned that the proper description of the microworld is given by quantum mechanics. Quantum mechanics gives more accurate results for chemical systems than any preceding model, however, it is very inexpressive for the classically thinking mind. One of the most used quantum mechanical concepts of the chemical bond is the valence bond theory^22^, among others^23, 24^, forming the bonds between atoms by overlap of the atomic orbitals. The valence bond theory complements the molecular orbital theory^25^, distributing pairs of electrons in bonding molecular orbitals delocalized over the system. In this work, in the spirit of the valence bond theory, we study correlations among the orbitals localised on individual atoms.

Indeed, studying the *two-orbital* correlation pattern in molecular systems in equilibrium gives us the hint that the correlations must be related to the chemical bonds: strong two-orbital correlations can be observed between the orbitals which are involved in the given bond^8, 10, 12–20, 26^. Simple covalent bonds formed by two atomic orbitals fit well into this *two-orbital correlation* picture. However, there are more complicated bonding scenarios with electrons shared by multiple atoms, in this case some true *multiorbital correlation* picture should be used^27^. (So far, multiorbital correlations were investigated by the use of the notion of two-orbital correlation only^7, 8, 10, 12–20, 26, 28^). The reason for this is twofold. On the one hand, such bonds, e.g., a delocalized ring in a benzene molecule, cannot be considered as a “sum” of two-orbital bonds, but a true multiorbital bond. On the other hand, in multiorbital systems, *hidden correlations* may occur, that is, there may be strong multiorbital correlation among the orbitals in a cluster even if the two-orbital correlations are weak.

In this work we provide the true multiorbital correlation theory, consisting of a framework for handling the structure of multiorbital correlations, a toolbox of true multiorbital correlation measures, and the definition together with an algorithm for the multiorbital correlation clustering. The presented theory significantly outgrows the multipartite entanglement theory^27^, on which it is based, namely, in the last three items mentioned just above. (The detailed construction is presented in the Supplementary). We adopt the principle that bonds are where the electrons can freely move among atoms, and this is reflected in the correlations of occupations of localised orbitals. Then we show illustrative results by investigating the multipartite correlations in several molecules showing multiorbital bonds. We give quantitative characterisation how well a bonding picture describes the chemical systems. We also illustrate that in the debated case of the dicarbon molecule, there is no well-defined multiorbital correlation clustering, which provides a reason for the ambiguous bonding picture^29–33^. This is not only the first true multiorbital correlation based study of the chemical bond, but also the first application of true multipartite correlation based techniques in physics.

We emphasise that the notions of correlations are basis dependent. We employed two basis sets in this study, standard STO-3G and STO-6G with optimised exponents, which latter provided HF energy close to HF/cc-pVTZ level of theory (see Methods section), together with the localisation procedure of Pipek and Mezey^34^ to produce atomic-like orbitals. All results discussed about the correlation structure of localised orbitals are understood with respect to this localisation. For the prototypical molecules which were considered for illustrating our theory, we have found that employing the minimal unchanged STO-3G basis set is sufficient for the description of bonding, and using a basis set closer to the Complete Basis Set limit have not changed the bonding picture. The results using optimised STO-6G basis set are presented in the main text, while the results using unchanged STO-3G basis set are also presented in the Supplementary for comparison.

We note that our work is not connected to previous works of de Giambiagi, Giambiagi and Jorge^35^ regarding generalised bond indices based on density-density correlation functions.

## Multiorbital correlations

For the quantum mechanical description of the molecule, we use the second quantized picture, that is, the Hilbert space of the electronic system is built up by the one-orbital Hilbert spaces, describing the occupation of the orbitals^36^. An orbital can be unoccupied, occupied with one electron of spin up or down, or doubly occupied by two electrons of spin up and down, resulting in four-dimensional one-orbital Hilbert spaces. In the Hilbert space formalism of quantum mechanics, any linear combination of orbitals is an orbital, however, the interpretation, or physical properties single out some of them. Correlations among orbitals are not invariant under such nonlocal (among-orbital) operations. In order that the correlations express some connection among local objects (atoms), it is necessary that the orbitals are *localised* on the atoms.

So, for the description of (the electronic system of) the *molecule*, we consider *m* localised, atomic-like orbitals. Let *M*, stands for “molecule”, denote the set of (the labels of) these orbitals. We aim at describing the correlations in an *L* ⊆ *M* set of orbitals (cluster). (If *L* = *M* then the correlations in the whole molecule is considered). In general, the *state* of the full electronic system of the cluster *L* can be described by the density operator^1, 37, 38^ *ρ*_*L*_. The *reduced state* of a (sub)cluster *X* ⊆ *L* can be described by the reduced density operator^1, 37, 38^ *ρ*_*X*_. If the cluster of orbitals *L* can be described by a state vector |*ψ*_*L*_〉 (for example, when a given eigenstate of the whole molecule is considered), then its density operator is of rank one, *ρ*_*L*_ = |*ψ*_*L*_〉〈*ψ*_*L*_|, called a *pure state*. Its reduced density operator is mixed (not of rank one) in general, which is the manifestation of the *entanglement* ^4^ of (sub)cluster *X* and the rest of the cluster *L*\*X*.

The correlation can be defined with respect to a split of the M set of the orbitals^27, 39^. Let *ξ* denote such a split, that is, a *partition* ^40^ *ξ* = {*X*_1_, *X*_2_, …, *X*_|*ξ*|_} ≡ X_1_|X_2_|…|X_|*ξ*|_, where the clusters *X* ∈ *ξ*, called *parts*, are disjoint subsets of the cluster *L*, and their union is the full cluster *L*. A natural comparison of partitions is the “refinement”: we say that partition *υ* is finer than partition *ξ*, if the parts of υ are contained in the parts of *ξ*. The set of the partitions of *L* is denoted with Π(*L*). (For illustrations, see Supplementary Fig. S1). The measure of correlation with respect to this split is the *ξ-correlation* ^27^,

1 $${C}_{\xi }({\rho }_{L}):=\sum _{X\in \xi }S({\rho }_{X})-S({\rho }_{L}).$$

Here *S*(*ρ*) = −tr *ρ* ln *ρ* is the von Neumann entropy^1, 37^. As a special case, the *i*|*j*-correlation

2 $${C}_{i|j}({\rho }_{\{i,j\}})=S({\rho }_{\{i,j\}})+S({\rho }_{\{j\}})-S({\rho }_{\{i,j\}})={I}_{i|j}({\rho }_{\{i,j\}}),$$

being the well-known (two-orbital) mutual information^1, 37, 41^, has already been used^7, 8, 10, 12–20, 26, 28^. The *ξ*-correlation is zero if the state is uncorrelated with respect to *ξ* (it can be written in a product form of reduced states of clusters *X* ∈ *ξ*); and nonzero otherwise, characterising the strength of the correlation among the parts *X* ∈ *ξ*. This comes from the information-geometrical meaning of this quantity: it characterises how “far” the state is from the states uncorrelated with respect to *ξ*. (For more details of the construction, see the Supplementary). Note that *C*_*ξ*_ is larger for finer partitions, (this is called *multipartite monotonicity* ^27^), it is zero for the trivial split $\xi =\top =\{L\}$, and it takes its maximum, *C*_⊥_, for the finest split *ξ* = ⊥ = {{*i*} | *i* ∈ *L*}. The latter quantity is also called *total correlation* ^42–46^,

3 $${C}_{{\rm{tot}}}({\rho }_{L}):={C}_{\perp }({\rho }_{L})=\sum _{i\in L}S({\rho }_{\{i\}})-S({\rho }_{L}).$$

(Note that if cluster *L* is described by a pure state, e.g., if *L* = *M*, then *S*(*ρ*_*L*_) = 0, and the correlation is entirely quantum entanglement^4, 5, 27^). It is easy to check the following *sum rule* ^46^, valid for any partition *ξ*,

4 $$\sum _{X\in \xi }{C}_{\perp ,X}({\rho }_{X})+{C}_{\xi }({\rho }_{L})={C}_{\perp }({\rho }_{L}),$$

that is, the total correlation is the sum of the total correlations inside the parts plus the correlation with respect to the partition.

We would also like to characterise the correlations in an overall sense, that is, without respect to a given partition. There are several ways of this^27^, here we consider two of them. Let us introduce the *k-partitionability correlation* and the *k-producibility correlation*, respectively,

5 $${C}_{k-{\rm{p}}{\rm{a}}{\rm{r}}{\rm{t}}}({\rho }_{L}):=\mathop{min}\limits_{\xi :|\xi |\ge k}{C}_{\xi }({\rho }_{L}),\,\,\,\,\,{C}_{k-{\rm{p}}{\rm{r}}{\rm{o}}{\rm{d}}}({\rho }_{L}):=\mathop{min}\limits_{\xi :{\rm{\forall }}X\in \xi ,|X|\le k}{C}_{\xi }({\rho }_{L}),$$

for 1 ≤ *k* ≤ |*L*|. These characterise two different (one-parameter-) notions of multiorbital correlations. The *k*-partitionability correlation is zero if the cluster can be split into at least *k* parts which are uncorrelated with one another, and the correlations are restricted only inside those parts; and nonzero otherwise, characterising the strength of this kind of correlation. In general, *C*_*k*-part_ increases with *k*, and it jumps after the number *k* of parts into which *L* can approximately be split. The *k*-producibility correlation is zero if the cluster *L* contains correlated (sub)clusters of size not larger than *k*; and nonzero otherwise, characterising the strength of this kind of correlation. In general, *C*_*k*-prod_ decreases with *k*, and it jumps at the size *k* of the largest part in the partition into which *L* can approximately be split. As special cases, *C*_|*L*|-part_ = *C*_1-prod_ = *C*_⊥_ grabs all the correlations, it is zero if there is no correlation at all in the cluster *L*, that is, its state is a product of the states of orbitals; and nonzero otherwise. On the other hand, *C*_2-part_ = *C*_(|*L*| − 1)-prod_ is sensitive only for the strongest correlations, it is nonzero if the cluster *L* is globally correlated, that is, its state is not a product of states of two (or more) clusters; and zero otherwise. (*C*_1-part_ = *C*_|*L*|-prod_ = ${C}_{\top }$ = 0. For other values of *k* there are no such coincidences among the partitionability and producibility correlations, however, the relation *C*_*k*-part_ ≥ *C*_(|*L*|−*k*+1)-prod_ holds. Also, the bounds *C*_*k*-part_ ≤ 2(*k* − 1) (ln4), C_*k*-prod_ ≤ 2(|L| − k) (ln4) hold. For more details, see the Supplementary).

The tools (1) and (5), despite being so simple, are proven very useful in a wide range of applications for the characterisation of multiorbital correlations in the electronic system of molecules. In the sequel, we show four of these applications. Illustrating these, we present numerical results for several prototypical molecules, namely benzene, pyrrole, borole, cyclobutadiene, furan, thiophene, and the sequence C_2_H_2*x*_ for *x* = 1, 2, 3 and C_2_.

## Applications

### Application 1: Molecule, formed by bonds

Our fundamental *principle* is that, in the equilibrium, *the bonds are almost uncorrelated with one another*, *and the orbitals involved in a bond are strongly* (*multiorbital*) *correlated*. Using the tools introduced above, we formulate this principle, and we demonstrate it for the aforementioned molecules.

An *ansatz* for the bond structure is given by a partition *β* = *B*_1_|*B*_2_|…|*B*_|*β*|_ ∈ Π(*M*) (*bond split*), representing the molecule as a set of *bonds* (represented by *B* ⊆ *M* sets of orbitals), together with some nonbonding orbitals (e.g., core orbitals or lone pairs, for those, |*B*| = 1). Then the *β*-correlation *C*_*β*_(*ρ*_*M*_), given in (1), characterising the correlation with respect to the ansatz *β*, expresses how well this ansatz describes the physical situation: the lower the *C*_*β*_ the better the ansatz from a *purely information-theoretical point of view*. The aim of this application is to find the bond split *β* (if exists) from *ab initio* data, without taking into account anything which can a priory be known about the bond structure in quantum chemistry. We call this *multiorbital correlation clustering*.

Since in a real electronic system one cannot expect such a simple ansatz to be exactly valid (*C*_*β*_ = 0), we actually pose the question, which ansatz *β* is the best choice for the description of the bonds from a *physical chemical point of view*. Being the best, however, is a delicate question. Note that, on the one hand, ${C}_{\top }$ = 0, for the trivial split $\beta =\top$, which takes the whole molecule to be one big bond. On the other hand, *C*_*β*_ grows with respect to the refinement of *β*, and takes its maximal value *C*_⊥_, the total correlation (3), for the finest split *β* = ⊥, which excludes nontrivial bonds. These extremal cases, obviously, do not give proper descriptions of the bond structure of a molecule, since, on the one hand, there can be clusters weakly correlated but not uncorrelated with the remaining part of the molecule, on the other hand, it is not allowed to neglect strong correlations. Instead of these, we have to be able to split the molecule into *weakly* correlated clusters consisting of *strongly* correlated orbitals. In order to grasp the meaning of the multipartite correlation clustering, we have to be able to decide about a given *ξ*, if it is a good ansatz, or it is worth considering a *ξ*′, which is “a bit” finer than *ξ*. That is, we have to investigate the difference *C*_*ξ*′_ − *C*_*ξ*_, where *ξ*′ is finer than *ξ*, and there is no other partition between them. We seek *β*, for which, *while ξ is coarser than β*, *this difference is small if ξ*′ *is coarser than β*, *but large*, *if ξ*′ *is not coarser than β*. If there exists such a *β*, then it is meaningful to consider the electronic system to be weakly correlated bonds consisting of strongly correlated orbitals, and this is described by *β*. (For the whole construction, see the Supplementary).

Here we face the problem that verifying that this definition holds for a given partition (calculating *S*(*ρ*_*X*_) for all clusters *X* ⊆ *M*, needed for the calculation of *C*_*ξ*_ for all partitions *ξ*) is numerically prohibitive. We can decrease the demands by successive refinement of the partitioning (bipartitioning of one cluster in each step) following the smallest increase in *C*_*ξ*_. One can show that if there exists a *β* satisfying the definition above, then the successive refinement goes through *β*, *C*_*ξ*_ increases slowly until *β*, and rapidly after *β*. (For the whole construction, see the Supplementary).

We can also have a hint for the path of the successive bipartitioning. Consider the *γ* = *G*_1_|*G*_2_|…|*G*_|*γ*|_ clustering based on the “connectivity” with respect to the two-orbital correlations (2). That is, the parts *G* ∈ *γ* are the sets of orbitals being the vertices of the connected components of the *two-orbital correlation graph* ^8, 28^. (This is the graph with vertices being the orbitals *i* ∈ *M* and with edges of weights *C*_*i*|*j*_(*ρ*_{*i*,*j*}_) above a threshold *T*_b_. We call this *two-orbital correlation clustering*). It is proven to be a good strategy to do the successive bipartitioning with respect to *γ*, that is, not to split apart the parts of *γ*. Following this strategy, one reaches *γ*; *C*_*ξ*_ increases rapidly after *γ*, but it is not sure that *C*_*ξ*_ increases slowly before *γ*. This is because of the possibility of the existence of *hidden correlations*, which is an interesting feature of the multiorbital setting. For example, there exist states in which all two-orbital correlations (2) are zero, but the states are correlated as a whole, that is, they cannot be written in a nontrivial product form (see the Supplementary). This means that if we follow the above strategy, then *C*_*ξ*_ may change rapidly before we reach *γ*. In this case, *β* does not equal to *γ*, but coarser than *γ*.

We have investigated the two-orbital and the multiorbital correlation clustering for the aforementioned molecules.

**Figure 1:**
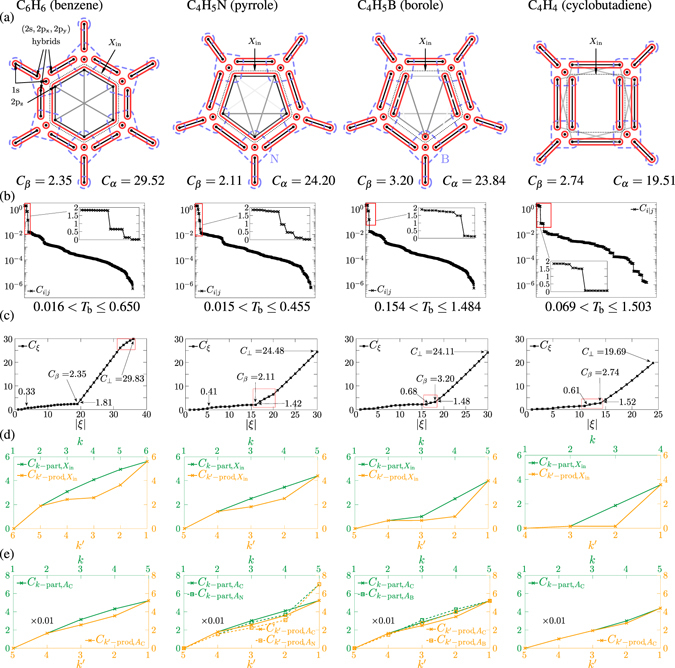
Partitioning and multipartite correlations for the benzene, pyrrole, borole and cyclobutadiene molecules. (**a**) Schematic view of the molecules: the dots represent atomic orbitals, the ones localised on an atom are encircled in dashed blue lines, this is the atomic split *α*, the ones strongly correlated with each other are encircled in solid red lines, this is the bond split *β*. Strength of edges represent two-orbital correlations (shaded by a logarithmic scale). The correlations *C*_*α*_ and *C*_*β*_ are also shown. (**b**) The distributions of the two-orbital correlations. The possible ranges of two-orbital correlation thresholds *T*_*b*_ are also shown. (**c**) The *C*_*ξ*_ tendencies of the successive bipartitioning. The humps arising from the bipartitioning of multiorbital correlated clusters are indicated with red frames. The maximal step before *β* and the minimal step following *β* are also shown. (**d**) The correlations ${C}_{k-\text{part},{X}_{{\rm{in}}}}$, ${C}_{k-\text{prod},{X}_{{\rm{in}}}}$ for the inner bonding (2p_z_) orbitals, contained in *X*_in_. (**e**) The correlations *C*_*k*-prod,*A*_, *C*_k-part,*A*_ for selected atoms A. (The numerical values of the correlation measures are given in units of ln4).

**Figure 2:**
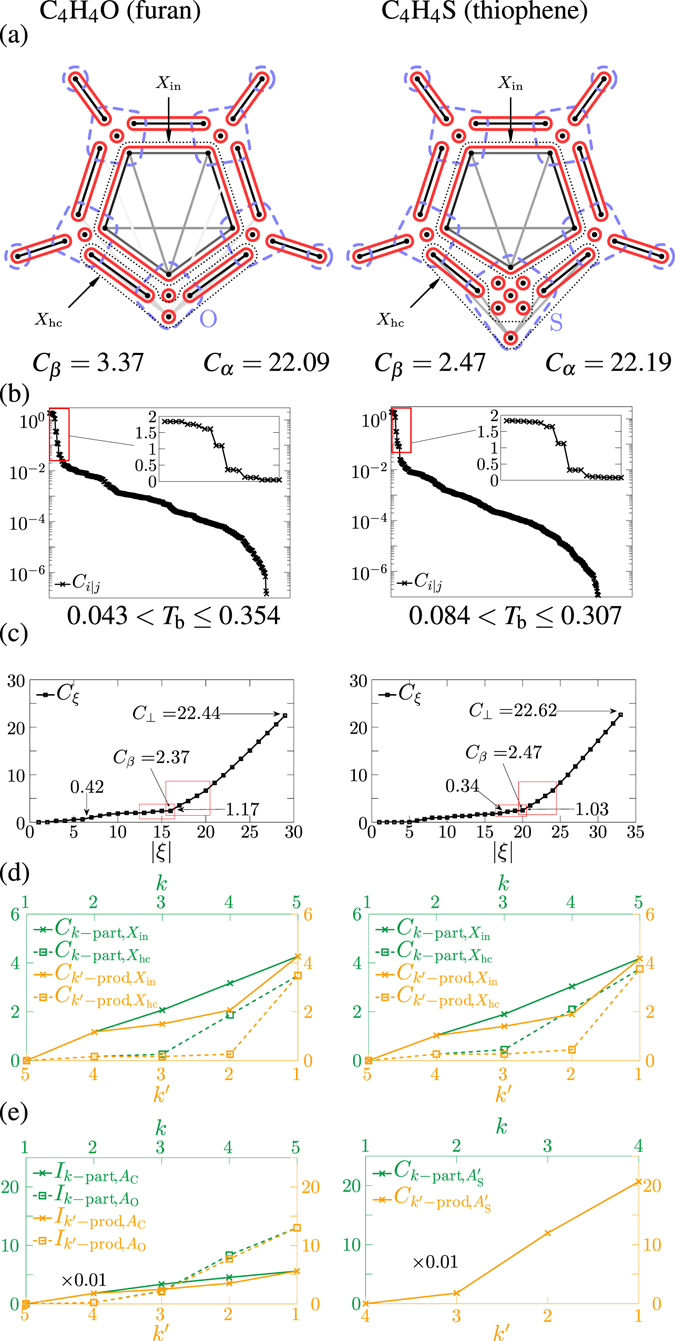
Partitioning and multipartite correlations for the furan and thiophene molecules. The same types of data are shown as in Fig. 1. (**d**) The correlations for the orbitals participated in the hyperconjugative interaction, contained in *X*_hc_ are also shown. (**e**) For the thiophene, the correlations among the valence orbitals, contained in ${A}_{S}^{^{\prime} }$ are shown.

**Figure 3:**
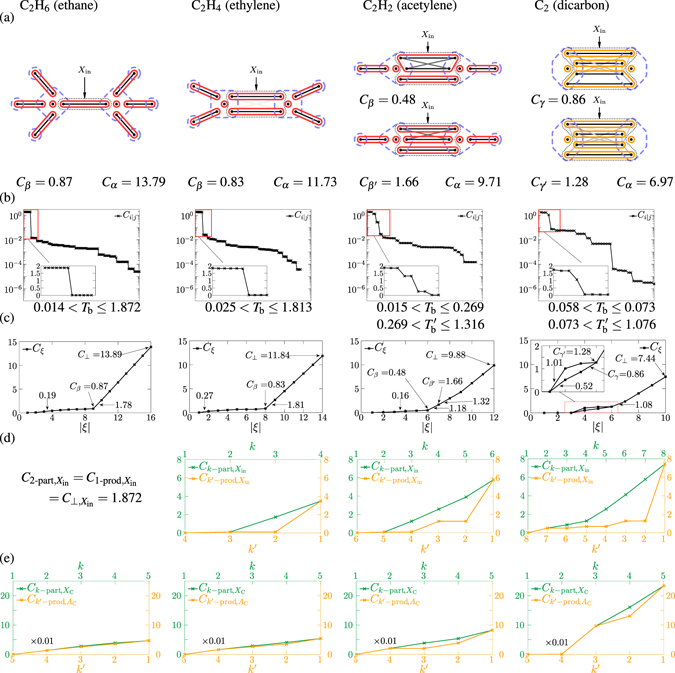
Partitioning and multipartite correlations for the C_2_H_2*x*_ molecules. The same types of data are shown as in Fig. 1.

The two-orbital correlations are drawn by different shades of grey lines in subfigures (a) of Figs 1, 2 and 3. The two-orbital correlation clusterings γ are based on the appropriate threshold values *T*_b_. The distributions of two-orbital correlations, and the possible two-orbital correlation thresholds *T*_b_ leading to the known bond structure in the given cases are shown in subfigures (b). For C_2_H_2_, there is a much wider range for ${T}_{{\rm{b}}}^{^{\prime} }$, leading to triple bond in *γ*′, than for *T*_b_, leading to double bond in *γ*, and for C_2_, there is a much wider range for ${T}_{{\rm{b}}}^{^{\prime} }$, leading to quadruple bond in *γ*′, than for *T*_b_, leading to triple bond in *γ*. A drawback of the two-orbital correlation clustering method is that, although the two-orbital correlation (2) is bounded by 0 ≤ *C*_*i*|*j*_ ≤ 2(ln4) uniformly, a uniform threshold covering all the cases is contained in a quite narrow range 0.269(ln4) < *T*_b_ ≤ 0.307(ln4). The reason for this is that an orbital seems to be *forced to share its* (*two-orbital*-)*correlations* among the ones strongly correlated with it, which may be a manifestation of the *monogamy of entanglement* ^47^ *in correlations*. (Different thresholds for the different cases may be obtained based on the separation of the correlation scales, however, this leads to a bond-interpretation rather arbitrary).

The multiorbital correlation clustering *β*, determined by the use of the method described above, is drawn by solid red lines in subfigures (a) of Figs 1, 2 and 3. The values of *C*_*ξ*_ during the successive bipartitioning are shown in subfigures (c). In the cases of the cyclic molecules (Figs 1 and 2) we could find a well-defined *β* bond split, after which the value of *C*_*ξ*_ jumps about at least twice as large as the maximal step before that. Note that in certain positions, some humps are observed in the tendencies *C*_*ξ*_ (designated with red rectangles). These are the effects of correlated clusters of size more than two: When the successive bipartitioning reaches such a cluster, following the first large step, smaller steps become possible, leading to this concave behaviour. Such humps are coming from two origins. The more characteristic one is the cluster of the inner bonding 2p_z_ orbitals (denoted with *X*_in_ in the figures). In the cases of *borole* and *cyclobutadiene*, these humps can be found directly before β, they are not steep enough to keep these orbitals together during the successive bipartitioning, contrary to those in the cases of *benzene*, *pyrrole*, *furan* and *thiophene*, when these humps can be found after *β*. In this way we can distinguish between aromaticity and antiaromaticity. On the other hand, in the cases of *furan* and *thiophene* (Fig. 2), there is an additional weaker multiorbital correlated structure in each case besides the aromatic rings, due to the hyperconjugative interaction of the lone pair with the adjacent *σ*-bonds^19^ (denoted with *X*_hc_ in the figures). The correlations in them are not strong enough to keep these orbitals together during the successive bipartitioning. (The almost uniform increase after *β* comes from the bipartitioning of the two-orbital clusters). In the cases of the C_2_H_2*x*_ sequence (Fig. 3), it can be seen how the correlation picture becomes more and more fuzzy. Interestingly, for the case of C_2_H_2_, investigating the tendency *C*_*ξ*_ during the successive bipartitioning in subfigure (c), one can see that it changes significantly at the partition leading to a double bond *β* (=*γ*), and there is a much less significant change at the partition leading to a triple bond *β*′ (=*γ*′). For bipartite correlation clustering, *γ*′ were more plausible than *γ*, however, here *β* seems to be more plausible than *β*′. This is indeed a very interesting observation, which might be to some extent an indication of hidden correlation (*β* is coarser than *γ*′). Note however, that despite not being divided in two in our multiorbital correlation point of view, the four-orbital bond does not contradict the classical picture of triple-bonded C_2_H_2_, as it contains four electrons. For the case of C_2_, investigating the tendencies *C*_*ξ*_ during the successive bipartitioning in subfigure (c), one cannot give a well-defined bond split *β* besides the 1 + 8 + 1-orbital partition, because of the high multiorbital correlation of the eight orbitals. (The splits *γ* and *γ*′, given by the two-orbital correlation clustering, are drawn by solid orange lines in subfigure (a). In subfigure (c), we show two different paths of the successive refinement in the partitioning of the eight bonding orbitals, the upper one shows a more significant hump, while the lower one leads through the triple bond *γ*). That is, according to our observations, there exists no well-defined multiorbital correlation clustering. The bonding situation in the multireference C_2_ is well known as a long-standing puzzle, and several bond orders have been suggested, including the extensively debated quadruple bond^29–33^. In spite of the fact that four strong two-orbital correlations have been found, from the reason mentioned above, we cannot give a decisive multiorbital correlation answer on the bond order of C_2_.

### Application 2: Bonds, formed by orbitals

The bonds, that is, the highly correlated clusters, given by the parts *B* ∈ *β*, are identified in the previous point. Now, we can investigate the correlations inside the bonds *B* ∈ *β*. For this purpose, we use the *k*-partitionability and k-producibility correlation *C*_*k*-part,*B*_ and *C*_*k*-prod,*B*_, respectively, (see (5)), both of them are considered with respect to the splits Π(*B*).

The results are again summarised in Figs 1, 2 and 3. For the two-orbital bonds *B* = {*i*, *j*} ∈ *β*, the important quantities boil down to the two-orbital correlation (2), *C*_2-part,{*i*,*j*}_ = *C*_1-prod,{*i*,*j*}_ = *C*_*i*|*j*_. Its magnitude can be read off from subfigures (b). More interesting is the case of bonds consisting of more than two orbitals. The 2p_z_ orbitals (contained in *X*_in_ ⊂ *M*) in the cases of *benzene*, *pyrrole*, *furan* and *thiophene* form aromatic bond, and in the cases of *borole* and *cyclobutadiene* do not. This can be seen in the full increasing and decreasing tendencies ${C}_{k-\text{part},{X}_{{\rm{in}}}}$ and ${C}_{k-\text{prod},{X}_{{\rm{in}}}}$, shown in subfigures (d): on the one hand, ${C}_{2-\text{part},{X}_{{\rm{in}}}}$ = ${C}_{(|{X}_{{\rm{in}}}|-1)-\mathrm{prod},{X}_{{\rm{in}}}}$ is high in the four aromatic cases, that is, the orbitals in *X*_in_ cannot be split even into two parts, and, accordingly, the greatest part is of size |*X*_in_|; on the other hand, ${C}_{2-\text{prod},{X}_{{\rm{in}}}}$ and ${C}_{2-\text{part},{X}_{{\rm{in}}}}$, respectively, ${C}_{3-\text{part},{X}_{{\rm{in}}}}$ are low in the two antiaromatic cases, that is, the orbitals in *X*_in_ can be split into parts of size at most 2, their number are 2, respectively 3. So we can distinguish between aromaticity and antiaromaticity also in this way. The orbitals participated in the hyperconjugative interaction in *furan* and *thiophene* (contained in *X*_hc_ ⊂ *M*) show weaker correlation than the ones in the aromatic ring. In the cases of the C_2_H_2*x*_ sequence, the orbitals participating in the bonds between the carbon atoms (contained in *X*_in_) are getting more and more multiorbital-correlated. For C_2_H_4_, ${C}_{2-\text{part},{X}_{{\rm{in}}}}$ and ${C}_{2-\text{prod},{X}_{{\rm{in}}}}$ are near zero, that is, the two two-orbital bonds can be considered independent. For C_2_H_2_, ${C}_{2-\text{part},{X}_{{\rm{in}}}}$ and ${C}_{4-\mathrm{prod},{X}_{{\rm{in}}}}$ are negligibly low, while ${C}_{3-\text{part},{X}_{{\rm{in}}}}$ is significant, leading again to a double-bond, containing a four-orbital one. For C_2_, ${C}_{2-\text{part},{X}_{{\rm{in}}}}$, although being relatively low, does not seem to be completely negligible. In the latter two cases, we can see now from a local point of view, which was proposed in the previous Section from a global point of view, that *X*_in_ is not divided *completely* into independent bonds.

### Application 3: Molecule, formed by atoms

An *atom* is now represented by an *A* ⊆ *M* set of orbitals, where the orbitals *i* ∈ *A* are the ones localised on the given atom. The molecule can be considered as a set of atoms, this can be represented by the split *α* = *A*_1_|*A*_2_|…|*A*_|*α*|_ ∈ Π(*M*) (*atomic split*) of the molecule. Here an important quantity is the *α*-correlation *C*_*α*_(*ρ*_*M*_), and the *α*-coarsened *k*-partitionability and *k*-producibility correlations *C*_*k*-part,*α*_ (*ρ*_*M*_), *C*_*k*-prod,*α*_ (*ρ*_*M*_). These characterise the different aspects of the correlations with respect to the atomic split *α*.

The atomic split *α* for the aforementioned molecules are drawn by dashed blue lines in subfigures (a) of Figs 1, 2 and 3. The values of C_α_ are also shown. Calculating *C*_*k*-part,*α*_ (*ρ*_*M*_) and *C*_*k*-prod,*α*_ (*ρ*_*M*_) is infeasible, due to the large density matrices of high entropy, however, note that we already have the largest members of these hierarchies, since *C*_|*α*|-prod,*α*_ (*ρ*_*M*_) = *C*_1-prod,*α*_ (*ρ*_*M*_) = *C*_*α*_ (*ρ*_*M*_). The value of this is near *C*_⊥_(*ρ*_*M*_), that is, as can be expected, the atoms are strongly correlated with one another in the molecules.

### Application 4: Atoms, formed by orbitals

The orbitals localised on given atoms are collected in the parts *A* ∈ *α* in the previous point. Now, we can investigate the correlations in the atoms *A* ∈ *α*. For this purpose, we use the *k*-partitionability and k-producibility correlation *C*_*k*-part,*A*_ and *C*_*k*-prod,*A*_, respectively, (see (5)), both of them are considered with respect to the splits Π(*A*).

We have investigated the nontrivial (non-H) atoms in the aforementioned molecules. Although not all the positions of the C atoms are equivalent in the molecules, the correlation measures take roughly the same values for those. The full increasing and decreasing tendencies *C*_*k*-part,*A*_ (*ρ*_*A*_) and *C*_*k*-prod,*A*_ (*ρ*_*A*_) are shown in subfigures (e). Note that the values of these are usually smaller than the correlations in the bonds, by about two orders of magnitude. In the sequence C_2_H_4_, C_2_H_2_ and C_2_ we can also see, how the increase of the multiorbital correlations leads to more and more strong correlations among the orbitals localised on the same C atom. The hyperconjugative interaction leads to the same results on the O and S atoms in furan and thiophene.

### Remarks on the applications

Having the results of all the four applications in hand, we can now observe how the sum rule (4) works. In the first two applications, when we considered the *β* bond split, ∑_*B*∈*β*_ *C*_⊥,*B*_ (*ρ*_*B*_) was large and *C*_*β*_ (*ρ*_*M*_) small; while in the second two applications, when we considered the *α* atomic split, ∑_*A*∈*α*_ *C*_⊥,*A*_ (*ρ*_*A*_) was small and *C*_*α*_ (*ρ*_*M*_) large, and these are connected by the sum rule (4) as

6 $${C}_{\perp }({\rho }_{M})=\sum _{A\in \alpha }{C}_{\perp ,A}({\rho }_{A})+{C}_{\alpha }({\rho }_{M})=\sum _{B\in \beta }{C}_{\perp ,B}({\rho }_{B})+{C}_{\beta }({\rho }_{M}).$$

Based on these, we can consider the molecule as the weakly correlated set of strongly correlated bonds, or the strongly correlated set of weakly correlated atoms. Note that this holds for the equilibrium structure, which is the only one considered here. If the internuclear distances are altered, which is a method for the investigation of bond-formation^12, 14–16, 20^, we expect that the above picture is altered accordingly, however, the sum rule (6) holds with altered numerical values.

On the other hand, we may give a definition of the molecule from a correlation point of view: the orbitals *M* form a molecule, if there exists no nontrivial partition which is coarser than *α*, describing the atoms, and *β*, describing the bonds, that is, $\alpha \vee \beta =\top$. (In these cases, intermolecular bonds do not appear in *β*).

## Conclusions and outlook

We have presented a novel theory of the chemical bond which is inspired by quantum information theory and based on multiorbital correlations. Contrary to the literature, where only two-orbital notions were considered, we have invented and used true multiorbital notions. Illustrating the use of this theoretical toolbox, we have investigated several small prototypical molecules and showed how in a black-box manner the bonding picture of a molecule naturally comes out from the multiorbital correlations of occupations of localised atomic-like orbitals. We have identified the bonds with the strongly correlated clusters, and characterised quantitatively how well a given bonding picture describes these molecules. Our tools are, e.g., able to distinguish between aromaticity and antiaromaticity in cyclic conjugated systems. On the example of the sequence C_2_H_2_, C_2_H_4_, and C_2_H_6_, we have seen that the increase of wide-range multiorbital correlations results in the decrease of the well-posedness of multiorbital correlation clustering. In the extreme case of C_2_, this leads to the nonexistence of a well-defined multiorbital correlation clustering, which provides a reason for the debated bonding picture.

We would like to emphasise again that the treatment in terms of true multiorbital correlations seems to be a very natural point of view in the investigation of bonding among more than two atoms. The multiorbital correlation based quantities have their statistical meaning on their own right, and we have already seen several of their applications. However, it would be interesting to relate them to other standard quantities in quantum chemistry, quantifying, e.g., bond strength or aromaticity. Besides aromaticity, this treatment may find applications also in multicenter transition metal cluster chemistry.

We have seen how the multiorbital correlations characterise the chemical bonds, if the orbitals are localised. We note, however, that the theory can also be applied to any (orthonormalized) sets of orbitals, then it characterises the correlations among those orbitals. These, of course, do not have to be related to the chemical bonds, but may be related to other chemical properties.

From the point of view of theoretical power and beauty, the multiorbital correlation theory provides a much more natural and flexible treatment for multiorbital situations than using only two-orbital correlations, done in preceding works. An example supporting this is given by the (6) application of the sum rule (4). Contrary to this, a treatment based only on two-orbital correlations is theoretically hard to grasp, due to monogamy-like issues of correlations in quantum systems. This is why the notion of hidden correlations is not well-defined, and to formulate a clear-cut (quantitative and/or operative) definition is an open question.

## Methods

For the numerical results shown in this paper we have performed calculations using the quantum chemistry version of the *density matrix renormalization group* (QC-DMRG) algorithm^36, 48–56^. We have controlled the numerical accuracy using the *dynamic block state selection* (DBSS) approach^36, 44, 57^ and the maximum number of block states varied in the range of 500–4000 for an a priory set quantum information loss threshold value *χ* = 10^−6^. The ordering of molecular orbitals along the one-dimensional topology of the DMRG was optimised using the Fiedler approach^10, 15^ and the active space was extended dynamically based on the *dynamically extended active space* (DEAS) procedure^6, 36^. We have used DMRG to obtain the optimised MPS wavefunction, which was then used to construct the reduced density matrices, from which the correlation measures (1) and (5) were calculated.

Geometries have been optimised at HF/cc-pVTZ level of theory which yielded sufficient geometries in accordance with higher level methods. To obtain the localised atomic orbitals for the DMRG procedure, we first optimised the exponents of the STO-6G basis set using the MRCC program^58–60^ which approach resulted in similar HF energy to the cc-pVTZ basis set result within 10^−2^ Hartree. Then we used the Pipek-Mezey procedure^34^ implemented in MOLPRO^61^ Version 2010.1, with tight threshold 10^−12^, and minimised the number of atomic orbitals contributed to each localised orbitals. All localised orbitals have been used in the DMRG procedure thus, as a result, we have carried out calculations at the FCI limit for all molecules. Then the results close to the FCI limit have been analysed in the paper^19^. We note that we also calculated all results using HF/STO-3G geometry and localised STO-3G orbitals, with literature value exponents, and found neglectable difference compared to the results presented in the manuscript. (These results are presented in the Supplementary for comparison). This suggests that our analysis is very robust in general. We note that this robustness is not entirely surprising. Mayer has shown^62–64^ that extracting chemical information from molecular wavefunctions such as bond orders and valence indices could also be obtained using only STO-3G basis set.

The datasets generated during and/or analysed during the current study are available from Sz.Sz. on reasonable request.

## Electronic supplementary material

The correlation theory of the chemical bond–supplementary material

## References

[1] Wilde, M. M. *Quantum Information Theory* (Cambridge University Press 2013).

[2] Nielsen, M. A. & Chuang, I. L. *Quantum Computation and Quantum Information* 1 edn (Cambridge University Press, 2000).

[3] Amico L, Fazio R, Osterloh A, Vedral V (2008). Entanglement in many-body systems. Rev. Mod. Phys..

[4] Horodecki R, Horodecki P, Horodecki M, Horodecki K (2009). Quantum entanglement. Rev. Mod. Phys..

[5] Modi K, Paterek T, Son W, Vedral V, Williamson M (2010). Unified view of quantum and classical correlations. Phys. Rev. Lett..

[6] Legeza Ö, Sólyom J (2003). Optimizing the density-matrix renormalization group method using quantum information entropy. Phys. Rev. B.

[7] Legeza Ö, Sólyom J (2006). Two-site entropy and quantum phase transitions in low-dimensional models. Phys. Rev. Lett..

[8] Rissler J, Noack RM, White SR (2006). Measuring orbital interaction using quantum information theory. Chemical Physics.

[9] Pipek J, Nagy I (2009). Measures of spatial entanglement in a two-electron model atom. Phys. Rev. A.

[10] Barcza G, Legeza Ö, Marti KH, Reiher M (2011). Quantum-information analysis of electronic states of different molecular structures. Phys. Rev. A.

[11] McKemmish, L. K., McKenzie, R. H., Hush, N. S. & Reimers, J. R. Quantum entanglement between electronic and vibrational degrees of freedom in molecules. *The Journal of Chemical Physics* **135** (2011). 10.1063/1.3671386 22225147

[12] Boguslawski K, Tecmer P, Barcza G, Legeza Ö, Reiher M (2013). Orbital entanglement in bond-formation processes. Journal of Chemical Theory and Computation.

[13] Kurashige Y, Chan GK-L, Yanai T (2013). Entangled quantum electronic wavefunctions of the Mn_4_CaO_5_ cluster in photosystem II. Nature Chemistry.

[14] Mottet M, Tecmer P, Boguslawski K, Legeza Ö, Reiher M (2014). Quantum entanglement in carbon-carbon, carbon-phosphorus and silicon-silicon bonds. Phys. Chem. Chem. Phys..

[15] Fertitta E, Paulus B, Barcza G, Legeza Ö (2014). Investigation of metal-insulator-like transition through the *ab initio* density matrix renormalization group approach. Phys. Rev. B.

[16] Duperrouzel C (2015). A quantum informational approach for dissecting chemical reactions. Chemical Physics Letters.

[17] Boguslawski K, Tecmer P (2015). Orbital entanglement in quantum chemistry. International Journal of Quantum Chemistry.

[18] Freitag L (2015). Orbital entanglement and casscf analysis of the ru-no bond in a ruthenium nitrosyl complex. Phys. Chem. Chem. Phys..

[19] Szilvási, T., Barcza, G. & Legeza, Ö. Concept of chemical bond and aromaticity based on quantum information theory. *arXiv [*physics.chem-*ph]* 1509.04241 (2015).

[20] Zhao Y (2015). Dissecting the bond-formation process of d^10^-metal–ethene complexes with multireference approaches. Theor. Chem. Acc..

[21] Lewis GN (1916). The atom and the molecule. Journal of the American Chemical Society.

[22] Shaik, S. S. & Hiberty, P. C. *A Chemist’s Guide to Valence Bond Theory* (Wiley 2007).

[23] Bader RFW, Stephens ME (1975). Spatial localization of the electronic pair and number distributions in molecules. Journal of the American Chemical Society.

[24] Daudel, R. Introduction to the loge theory. In Chalvet, O., Daudel, R., Diner, S. & Malrieu, J. P. (eds) *Localization and Delocalization in Quantum Chemistry: Volume I Atoms and Molecules in the Ground State*, 3–8 (Springer Netherlands, Dordrecht 1975).

[25] Fleming, I. *Molecular Orbitals and Organic Chemical Reactions: Reference Edition* (Wiley 2010).

[26] Murg V, Verstraete F, Schneider R, Nagy PR, Legeza Ö (2015). Tree tensor network state with variable tensor order: An efficient multireference method for strongly correlated systems. Journal of Chemical Theory and Computation.

[27] Szalay Sz (2015). Multipartite entanglement measures. Phys. Rev. A.

[28] Barcza G, Noack RM, Sólyom J, Legeza Ö (2015). Entanglement patterns and generalized correlation functions in quantum many-body systems. Phys. Rev. B.

[29] Shaik S (2012). Quadruple bonding in C_2_ and analogous eight-valence electron species. Nature Chemistry.

[30] Shaik S, Rzepa HS, Hoffmann R (2013). One molecule, two atoms, three views, four bonds?. Angewandte Chemie International Edition.

[31] Grunenberg J (2012). Quantum chemistry: Quadruply bonded carbon. Nature Chemistry.

[32] Frenking G, Hermann M (2013). Critical comments on “one molecule, two atoms, three views, four bonds”?. Angewandte Chemie International Edition.

[33] Zhong R, Zhang M, Xu H, Su Z (2016). Latent harmony in dicarbon between VB and MO theories through orthogonal hybridization of 3*σ*g and 2*σ*u. Chem. Sci..

[34] Pipek J, Mezey PG (1989). A fast intrinsic localization procedure applicable for abinitio and semiempirical linear combination of atomic orbital wave functions. The Journal of Chemical Physics.

[35] de Giambiagi MS, Giambiagi M, Jorge FE (1985). Bond index: relation to second-order density matrix and charge fluctuations. Theoretica chimica acta.

[36] Szalay Sz (2015). Tensor product methods and entanglement optimization for *ab initio* quantum chemistry. Int. J. Quantum Chem..

[37] Ohya, M. & Petz, D. *Quantum Entropy and Its Use*, 1 edn (Springer Verlag 1993).

[38] Araki H, Moriya H (2003). Equilibrium statistical mechanics of fermion lattice systems. Reviews in Mathematical Physics.

[39] Szalay Sz, Kökényesi Z (2012). Partial separability revisited: Necessary and sufficient criteria. Phys. Rev. A.

[40] Davey, B. A. & Priestley, H. A. *Introduction to Lattices and Order*, second edn (Cambridge University Press 2002).

[41] Adesso G, Bromley TR, Cianciaruso M (2016). Measures and applications of quantum correlations. Journal of Physics A: Mathematical and Theoretical.

[42] Lindblad G (1973). Entropy, information and quantum measurements. Communications in Mathematical Physics.

[43] Horodecki R (1994). Informationally coherent quantum systems. Physics Letters A.

[44] Legeza Ö, Sólyom J (2004). Quantum data compression, quantum information generation, and the density-matrix renormalization-group method. Phys. Rev. B.

[45] Legeza Ö, Gebhard F, Rissler J (2006). Entanglement production by independent quantum channels. Phys. Rev. B.

[46] Herbut F (2004). On mutual information in multipartite quantum states and equality in strong subadditivity of entropy. Journal of Physics A: Mathematical and General.

[47] Koashi M, Winter A (2004). Monogamy of quantum entanglement and other correlations. Phys. Rev. A.

[48] White SR, Martin RL (1999). *Ab initio* quantum chemistry using the density matrix renormalization group. The Journal of Chemical Physics.

[49] White SR (1992). Density matrix formulation for quantum renormalization groups. Phys. Rev. Lett..

[50] Marti KH, Reiher M (2010). The density matrix renormalization group algorithm in quantum chemistry. Zeitschrift für Physikalische Chemie.

[51] Zgid, D. & Nooijen, M. On the spin and symmetry adaptation of the density matrix renormalization group method. *The Journal of Chemical Physics* **128** (2008). 10.1063/1.2814150 18190185

[52] Kurashige, Y. & Yanai, T. High-performance *ab initio* density matrix renormalization group method: Applicability to large-scale multireference problems for metal compounds. *The Journal of Chemical Physics* **130** (2009). 10.1063/1.3152576 19548718

[53] Legeza, Ö., Rohwedder, T., Schneider, R. & Szalay, Sz. Tensor product approximation (DMRG) and coupled cluster method in quantum chemistry. In Bach, V. & Delle Site, L. (eds) *Many-Electron Approaches in Physics*, *Chemistry and Mathematics*, Mathematical Physics Studies, 53–76 (Springer International Publishing 2014).

[54] Wouters, S. & Van Neck, D. The density matrix renormalization group for *ab initio* quantum chemistry. *The European Physical Journal D* **68** (2014).

[55] Chan GK-L, Kesselman A, Nakatani N, Li Z, White SR (2016). Matrix product operators, matrix product states, and *ab initio* density matrix renormalization group algorithms. The Journal of Chemical Physics.

[56] Olivares-Amaya, R. *et al*. The ab-initio density matrix renormalization group in practice. *The Journal of Chemical Physics* **142** (2015). 10.1063/1.4905329 25612684

[57] Legeza Ö, Röder J, Hess BA (2003). Controlling the accuracy of the density-matrix renormalization-group method: The dynamical block state selection approach. Phys. Rev. B.

[58] Kállay, M. *et al*. MRCC, a quantum chemical program suite, version 2016-07-15 www.mrcc.hu. (2016).

[59] Rolik Z, Szegedy L, Ladjánszki I, Ladóczki B, Kállay M (2013). An efficient linear-scaling CCSD(T) method based on local natural orbitals. The Journal of Chemical Physics.

[60] Mester D, Csontos J, Kállay M (2015). Unconventional bond functions for quantum chemical calculations. Theoretical Chemistry Accounts.

[61] Werner, H. J., Knowles, P. J., Knizia, G., Manby, F. R. & Schütz, M. Molpro, version 2010.1, a package of *ab initio* programs, http://www.molpro.net (2002).

[62] Mayer I (1983). Charge, bond order and valence in the *AB initio* SCF theory. Chemical Physics Letters.

[63] Mayer I (2007). Bond order and valence indices: A personal account. Journal of Computational Chemistry.

[64] Mayer, I. *Bond Orders and Energy Components: Extracting Chemical Information from Molecular Wave Functions* (Taylor & Francis 2016).

