# Supplementary material for: The correlation theory of the chemical bond
Source: Sci Rep. 2017 May 22;7:2237. doi: 10.1038/s41598-017-02447-z (PMC5440380; doi:10.1038/s41598-017-02447-z)
Supplement: Supplementary file 1 — The correlation theory of the chemical bond–supplementary material [file 41598_2017_2447_MOESM1_ESM.pdf]

The correlation theory of the chemical bond – supplementary material

Szilárd Szalay,[1] Gergely Barcza,[1] Tibor Szilvási,[2,3] Libor Veis,[4] and Örs Legeza[1]
[1]Strongly Correlated Systems "Lendület" Research Group, Institute for Solid State Physics and Optics, MTA Wigner Research Centre for Physics, H-1121 Budapest, Konkoly-Thege Miklós út 29-33, Hungary
[2]Department of Chemical and Biological Engineering, University of Wisconsin-Madison, 1415 Engineering Drive, Madison, Wisconsin 53706, United States
[3]Department of Inorganic and Analytical Chemistry, Budapest University of Technology and Economics, H-1111 Budapest, Szent Gellért tér 4, Hungary
[4]J. Heyrovský Institute of Physical Chemistry, Academy of Sciences of the Czech Republic, CZ-18223 Prague, Czech Republic

I. MULTIPARTITE CORRELATIONS

Here we recall the two-level theory of multipartite correlations, introduced earlier for the investigation of multipartite entanglement[1]. Furthermore, we extend the construction with the formalism of restriction to subsystems and coarsening, and we also construct bounds and relations for the resulting correlation measures. Using these tools we formulate and solve the task of multipartite correlation clustering, that is, dividing the whole system into weakly correlated subsystems consisting of strongly correlated elementary subsystems.

A. Setting the stage.

Consider a quantum system composed of n elementary subsystems, labelled by natural numbers $L = \{1, 2, \dots, n\}$. For the quantum mechanical description of every elementary subsystem, a Hilbert space of uniform dimension d is used. These can form the whole system investigated, or only a subsystem of that. Note that, in the Supplementary, we give a general treatment.[2–5] In the case in which we use these tools in the main text, the elementary subsystems are the orbitals (or clusters of orbitals in coarsened cases), that is, we use the second quantized formalism there, contrary to the first quantized formalism, in which case the elementary subsystems are the electrons. (A dictionary is given in Table S1.) Note that, however, this general treatment works equally well in the first and the second quantized pictures, this is why we have chosen this way of presentation here. In the first quantized picture, the construction characterises the correlations of the, e.g., position (or spin or other degrees of freedom) of different distinguishable particles, and in the second quantized picture, the same construction characterises the correlations of the occupation of states with different, e.g., position (or spin or other degrees of freedom). We also note that in the case of fermionic particles, the occupations of the sites are given in terms of anticommuting operators. This leads to some difficulties when one uses tools working well in the distinguishable case. However, it has turned out that all what we need during the construction of our multipartite/multiorbital correlation clustering are working well if the situation is restricted to the physical subspace of the operator algebra,[6–8] consisting of parity-preserving operators. Since in the molecular-physical situations, considered in the main text, even the preservation of the particle number holds, the following construction can obviously be applied.

The *state* of the quantum system is represented by a normalised positive linear functional acting on the algebra of the observables. It is given by the density operator ϱ_L, which is a positive semidefinite operator of trace one, acting on the Hilbert space associated to the system.[2,4,5] By restricting the state to the subalgebra of a (not necessarily elementary) subsystem $X \subseteq L$, one can form the reduced density operator ϱ_X of the subsystem. An essential property of a quantum state is its *mixedness*. It can be characterised by the von Neumann entropy[2,5,9]

$$S(\varrho) = -\operatorname{tr} \varrho \ln \varrho. \tag{1}$$

One can also compare two quantum states in the sense of statistical distinguishability[10] by the Umegaki relative entropy[2,11,12] (or quantum Kullback-Leibler divergence)

$$D(\varrho||\omega) = \operatorname{tr} \varrho(\ln \varrho - \ln \omega). \tag{2}$$

These two functions are of central importance in quantum information theory,[3–5] and the whole construction we build here is based on them. Both of these functions are nonnegative,[2,11] and they have several beautiful properties, making them extremely useful, also in the cases in which we apply them in the sequel. Maybe the most important one is the *strong subadditivity* of the von Neumann entropy[2,6,13]

$$S(\varrho_{X\cup X'}) + S(\varrho_{X\cap X'}) \leq S(\varrho_X) + S(\varrho_{X'}). \tag{3a}$$

A special case for disjoint subsystems X and X' is the *subadditivity* of the von Neumann entropy,

$$S(\varrho_{X\cup X'}) \leq S(\varrho_X) + S(\varrho_{X'}). \tag{3b}$$

From the strong subadditivity, the so called *Araki-Lieb triangle inequality* of the von Neumann entropy[2,4,5,7,14] also follows

$$\big|S(\varrho_X) - S(\varrho_{X'})\big| \leq S(\varrho_{X\cup X'}). \tag{3c}$$

Maybe even more fundamental is the *monotonicity* of the relative entropy *with respect to state reduction*,[2,6,15–17] that is, for subsystems $Y \subseteq X$,

$$D(\varrho_X||\omega_X) \geq D(\varrho_Y||\omega_Y). \tag{4}$$

quantum information theory	quantum mechanics (distinguishable particles) (first quantized)	many-body quantum physics (second quantized)	quantum chemistry (Born-Oppenheimer appr.) (second quantized)
system	ensemble of particles	chain, lattice	electronic system of molecule
elementary subsystem	particle	site	orbital
composite subsystem	subensemble of particles	block/cluster (of sites)	cluster (of orbitals)

Supplementary Table S1. Dictionary. In the main text we follow mainly the quantum chemistry language, because of the illustrative chemical applications, while in this Supplementary we use the general quantum information theory language. Note that the elementary subsystems are always distinguishable in this treatment. We do not treat the correlations in the first quantized formalism of indistinguishable particles.

B. Level I correlations.

A split of the system into *parts* is given by the *partition* $\xi = \{X_1, X_2, \dots, X_{|\xi|}\} \equiv X_1|X_2|\dots|X_{|\xi|}$ of the labels L, that is, the $X \in \xi$ sets of labels are nonempty and disjoint, and their union is the whole L. The set of the partitions of L is denoted with $\Pi(L)$. The partitions are illustrated with small pictographs in Fig. S1 for $n = 2, 3, 4$ subsystems. For two partitions, υ is a *refinement*[18] of ξ, ("υ is *finer* than ξ", or, "ξ is *coarser* than υ") denoted with $\upsilon \preceq \xi$, if ξ can be obtained from υ by joining some (maybe none) of the parts of υ. That is,

$$\upsilon \preceq \xi \quad \overset{\text{def.}}{\Longleftrightarrow} \quad \forall Y \in \upsilon, \exists X \in \xi : \; Y \subseteq X. \tag{5}$$

The refinement relation is a partial order, and the set of partitions $\Pi(L)$ turns out to be a lattice[18]. The top and bottom elements are $\top = \{\{1, 2, \dots, n\}\} = \{L\}$ and $\bot = \{\{1\}, \{2\}, \dots, \{n\}\} \equiv 1|2|\dots|n$, respectively. Later we will also need the notion of being neighbours in the lattice. This is called covering relation.[18] For two partitions, ξ *covers* υ, denoted with $\upsilon \prec\!\!\cdot\; \xi$, if $|\upsilon| = |\xi| + 1$, while $\upsilon \prec \xi$, from which we can conclude that there exists exactly one part $X_* \in \xi$, for which there are exactly two parts $Y_{*1}, Y_{*2} \in \upsilon$, such that $X_* = Y_{*1} \cup Y_{*2}$; and all the other parts in ξ can also be found in υ. That is,

$$\upsilon \prec\!\!\cdot\; \xi \quad \Longleftrightarrow \quad \begin{cases} \upsilon \setminus \xi = \{Y_{*1}, Y_{*2}\} \in \Pi(X_*), & \text{and} \\ \xi \setminus \upsilon = \{X_*\} \in \Pi(X_*). \end{cases} \tag{6}$$

The covering relation is illustrated with arrows in Fig. S1 for $n = 2, 3, 4$ subsystems.

A quantum state ϱ_L is ξ-*uncorrelated*, that is, uncorrelated with respect to the partition ξ, if it can be written in a product form of the reduced states with respect to ξ. One can characterise to what extent a state is not ξ-uncorrelated. To this end, let the ξ-*correlation*[1], also called "among-the-clusters correlation information"[19] be defined as

$$C_\xi(\varrho_L) := \sum_{X \in \xi} S(\varrho_X) - S(\varrho_L), \tag{7}$$

with the reduced states ϱ_X. (For the finest $\bot$ split, this is also called "correlation information"[19], or "multipartite mutual information"[20], or "total correlation"[21,22], also considered by Lindblad[23] and used[24] to describe correlations within multipartite quantum systems.) For a bipartition $\xi = X_1|X_2 \equiv X_1|(L \setminus X_1)$, the quantity $C_{X_1|X_2}(\varrho_L) = S(\varrho_{X_1}) + S(\varrho_{X_2}) - S(\varrho_L) = I_{X_1|X_2}(\varrho_L)$ is the well-known (bipartite) mutual information.[2–5] The information-geometrical meaning of the ξ-correlation is also clarified[1,25], it expresses the minimal distinguishability (2) of a state from the set of ξ-uncorrelated states, the "relative entropy of ξ-correlation",

$$\min_{\omega_L \; \xi\text{-uncorr.}} D(\varrho_L \| \omega_L) = C_\xi(\varrho_L). \tag{8}$$

Note that because of the (3b) subadditivity of the von Neumann entropy, the ξ-correlation takes higher value for a finer split,

$$\upsilon \preceq \xi \quad \Longleftrightarrow \quad C_\upsilon \geq C_\xi. \tag{9}$$

This is called *multipartite monotonicity (of the first kind)*.[1]

Some remarks on the relation to entanglement[26–28] is also on place here. If the whole system can be described by a pure state $\varrho_L = |\psi_L\rangle\langle\psi_L|$, the (7) correlation with respect to the $X|(L \setminus X)$ split is just two times the entanglement entropy[3,4,29] of subsystem $X \subseteq L$, that is, $C_{X|(L\setminus X)}(\varrho_L) = 2S(\varrho_X)$, because of the Schmidt decomposition[3,4,30]. Generally speaking, pure states of classical systems are always uncorrelated. If a pure state of a quantum system is correlated, then this correlation is of quantum origin, and it is called *entanglement*. Then correlation measures for pure quantum states often lead to entanglement measures[1]. Mixed states of a classical system can be either correlated or uncorrelated. If a mixed state of a quantum system is correlated, this correlation can either be classical or it can contain also quantum entanglement[25,31]. For mixed states, entanglement measures can also be constructed[1,32].

C. Level II correlations.

We also need to consider a second level notion of uncorrelated states. This expresses that a state is uncorrelated with respect to at least one partition from a given set. If ξ-uncorrelated states are considered, then υ-uncorrelated states are automatically considered also for

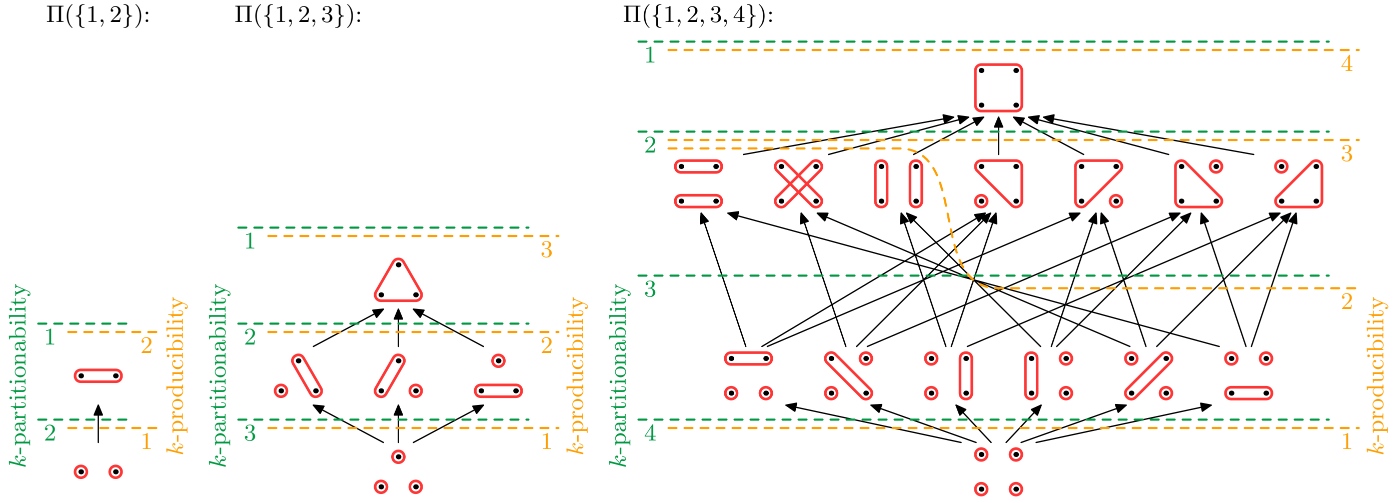

Supplementary Figure S1. Lattices of partitions for $n = 2, 3, 4$ subsystems. The covering is depicted with arrows from the finer to the coarser partition. The nonempty down-sets corresponding to the notions of k-partitionably uncorrelated and k-producibly uncorrelated are located under the dashed lines starting from the left and the right, respectively.

all $\upsilon \preceq \xi$, so it is natural to consider the nonempty *down-sets* of partitions[1] (also called *order ideal*). A nonempty $\boldsymbol{\xi} = \{\xi_1, \xi_2, \dots, \xi_{|\boldsymbol{\xi}|}\} \subseteq \Pi(L)$ set is a nonempty *down-set* if it contains every partition which is finer than its maximal elements.[18] This can be drawn by lines cutting the partition lattice into two parts in a way that the arrows cross these lines in one way only. The elements of the down-set are then the ones located under that line. Some of these cuttings are illustrated with dashed lines in Fig. S1 for $n = 2, 3, 4$ subsystems. Here we also have a natural partial order, being the standard set-theoretical inclusion among the nonempty down-sets, $\boldsymbol{\upsilon} \preceq \boldsymbol{\xi}$ if and only if $\boldsymbol{\upsilon} \subseteq \boldsymbol{\xi}$, and the set of nonempty down-sets of partitions is also a lattice[18]. Particular down-sets are the *principal ideals* $\downarrow\{\xi\} = \{\xi' \in \Pi(L) \mid \xi' \preceq \xi\}$, being the down-sets of partitions finer than or equal to a single ξ. Easy to check that $\downarrow\{\xi\} \preceq \downarrow\{\xi'\} \Leftrightarrow \xi \preceq \xi'$, so the Level I structure is enbedded into Level II in this way. (A dual way of this embedding is using the *principal filters* $\uparrow\{\xi\} = \{\xi' \in \Pi(L) \mid \xi \preceq \xi'\}$, which is a particular kind of *up-set* or *order filter*.)

A quantum state ϱ_L is $\boldsymbol{\xi}$*-uncorrelated*, if it is ξ-uncorrelated with respect to at least one $\xi \in \boldsymbol{\xi}$. One can characterise to what extent a state is not $\boldsymbol{\xi}$-uncorrelated. To this end, let the $\boldsymbol{\xi}$*-correlation*[1] be defined as

$$C_{\boldsymbol{\xi}}(\varrho_L) := \min_{\xi \in \boldsymbol{\xi}} C_\xi(\varrho_L). \tag{10}$$

The information-geometrical meaning of the $\boldsymbol{\xi}$-correlation is also clarified[1], it expresses the minimal distinguishability (2) of a state from the set of $\boldsymbol{\xi}$-uncorrelated states,

$$\begin{aligned} \min_{\omega_L\ \boldsymbol{\xi}\text{-uncorr.}} D(\varrho_L \| \omega_L) &= \min_{\forall \xi \in \boldsymbol{\xi}} \min_{\omega_L\ \xi\text{-uncorr.}} D(\varrho_L \| \omega_L) \\ &= \min_{\forall \xi \in \boldsymbol{\xi}} C_\xi(\varrho_L) = C_{\boldsymbol{\xi}}(\varrho_L), \end{aligned} \tag{11}$$

see (8). Note that because of the multipartite monotonicity (9) of the ξ-correlation (7), it is sufficient to calculate the minimum over the maximal elements of $\boldsymbol{\xi}$ in the $\boldsymbol{\xi}$-correlation (10), that is, $C_{\boldsymbol{\xi}}(\varrho_L) = \min_{\xi \in \max \boldsymbol{\xi}} C_\xi(\varrho_L)$. In particular, $C_{\downarrow\{\xi\}} = C_\xi$ for the principal ideal $\downarrow\{\xi\}$. Note on the other hand that, because of the multipartite monotonicity (9) of the ξ-correlation, the $\boldsymbol{\xi}$-correlation takes higher value for a smaller nonempty down-set,

$$\boldsymbol{\upsilon} \preceq \boldsymbol{\xi} \quad \Longleftrightarrow \quad C_{\boldsymbol{\upsilon}} \geq C_{\boldsymbol{\xi}}. \tag{12}$$

This is called *multipartite monotonicity (of the second kind)*[1].

D. k-partitionability and k-producibility.

So far we introduced different kinds of uncorrelated states, and we characterised to what extent a state is not uncorrelated in the different ways. This construction actually led to different *notions* of correlations. For our goals it is enough to consider some special ones of them.

For $k = 1, 2, \dots, n$, a state is k*-partitionably uncorrelated*, if it can be written in the product form of density matrices of (at least) k subsystems. That is, they are $\boldsymbol{\mu}_k$-uncorrelated for the nonempty down-set $\boldsymbol{\mu}_k$ containing all the partitions μ in which the *number ($|\mu|$) of the parts* is greater than or equal to k,[1]

$$\boldsymbol{\mu}_k = \big\{\mu \in \Pi(L) \bigm| |\mu| \geq k\big\}. \tag{13a}$$

(This is related to the natural gradation of the lattice of partitions $\Pi(L)$.) These form a chain (a completely ordered set), $\{\bot\} = \boldsymbol{\mu}_n \preceq \dots \preceq \boldsymbol{\mu}_{k+1} \preceq \boldsymbol{\mu}_k \preceq \dots \preceq \boldsymbol{\mu}_1 = \Pi(L)$. ($k$-partitionability is related to the k-separability in the theory of multipartite entanglement[33–35]: a mixed state is said to be k-separable, if it can be written as the

convex mixture of k-partitionably uncorrelated states. We would call them k-partitionably separable in an extended nomenclature, describing correlation and entanglement.) The cuttings corresponding to the nonempty down-sets $\boldsymbol{\mu}_k$ are illustrated with dashed green lines in Fig. S1 for $n = 2, 3, 4$ subsystems.

For $k = 1, 2, \dots, n$, a state is *k-producibly uncorrelated*, if it can be written in the product form of density matrices of subsystems of size at most k. That is, they are $\boldsymbol{\nu}_k$-uncorrelated for the nonempty down-set $\boldsymbol{\nu}_k$ containing all the partitions ν in which the *sizes* ($|N|$) *of the parts* $N \in \nu$ are less than or equal to k,[1]

$$\boldsymbol{\nu}_{k'} = \big\{\nu \in \Pi(L) \;\big|\; \forall N \in \nu : |N| \leq k'\big\}. \tag{13b}$$

(This is related to a "dual view" of the lattice of partitions $\Pi(L)$.) These form a chain, $\{\perp\} = \boldsymbol{\nu}_1 \preceq \dots \preceq \boldsymbol{\nu}_{k-1} \preceq \boldsymbol{\nu}_k \preceq \dots \preceq \boldsymbol{\nu}_n = \Pi(L)$. ($k$-producibility was originally introduced for the studying of multipartite entanglement[34,36,37], here we use an analogue of that for correlation: a mixed state is said to be k-producible, if it can be written as the convex mixture of k-producibly uncorrelated states. We would call them k-producibly separable in an extended nomenclature, describing correlation and entanglement.) The cuttings corresponding to the nonempty down-sets $\boldsymbol{\nu}_k$ are illustrated with dashed orange lines in Fig. S1 for $n = 2, 3, 4$ subsystems.

One can characterise to what extent a state is not k-partitionably and k-producibly uncorrelated by the use of the $\boldsymbol{\mu}_k$- and $\boldsymbol{\nu}_k$-correlation (10). In this case we call that *k-partitionability correlation* and *k-producibility correlation*, respectively,

$$C_{k\text{-part}}(\varrho_L) := C_{\boldsymbol{\mu}_k}(\varrho_L) \equiv \min_{\mu \in \boldsymbol{\mu}_k} C_\mu(\varrho_L), \tag{14a}$$

$$C_{k\text{-prod}}(\varrho_L) := C_{\boldsymbol{\nu}_k}(\varrho_L) \equiv \min_{\nu \in \boldsymbol{\nu}_k} C_\nu(\varrho_L). \tag{14b}$$

That is, for an n-partite system, we have two groups of n functions measuring correlation. Because of the multipartite monotonicity (12), the k-partitionability correlation takes lower value for a smaller k, and the k-producibility correlation takes higher value for a smaller k,

$$k \leq k' \quad \Longleftrightarrow \quad C_{k\text{-part}} \leq C_{k'\text{-part}}, \tag{15a}$$

$$k \leq k' \quad \Longleftrightarrow \quad C_{k\text{-prod}} \geq C_{k'\text{-prod}}. \tag{15b}$$

Note that $\boldsymbol{\mu}_1 = \boldsymbol{\nu}_n = \Pi(L)$, so $C_{1\text{-part}} = C_{n\text{-prod}} = 0$; $\boldsymbol{\mu}_2 = \boldsymbol{\nu}_{n-1}$, so $C_{2\text{-part}} = C_{(n-1)\text{-prod}}$; $\boldsymbol{\mu}_n = \boldsymbol{\nu}_1 = \{\perp\}$, so $C_{n\text{-part}} = C_{1\text{-prod}} = C_{\{\perp\}} = C_\perp$. There are no such coincidences for other values of k in general, however, $\boldsymbol{\mu}_k \preceq \boldsymbol{\nu}_{n-k+1}$ (because, for any partition $\xi \in \Pi(L)$, the relation $|X| \leq n - (|\xi| - 1)$ holds for all $X \in \xi$ parts), so

$$C_{k\text{-part}} \geq C_{(n-k+1)\text{-prod}}, \tag{16}$$

because of the multipartite monotonicity (12).

E. Correlations in subsystems and coarsening.

Until this point, we considered the correlation measures (7), (10) and (14) in a system $L = \{1, 2, \dots, n\}$ of elementary subsystems by the use of the partitions $\xi = \{X_1, X_2, \dots, X_{|\xi|}\} \equiv X_1|X_2|\dots|X_{|\xi|} \in \Pi(L)$. There are two ways for making these concepts more flexible.

First, we would like to characterise the correlations in a (nonempty) subsystem $L' \subseteq L$. Then for Level I, obviously, we have the ξ'-correlation for $\xi' \in \Pi(L')$ with the same definition (7). For Level II, we also have the $\boldsymbol{\xi}'$-correlation for a nonempty down-set $\boldsymbol{\xi}' \subseteq \Pi(L')$ with the same definition (10). For the k-partitionability and k-producibility correlations, we have to denote the restriction for subsystem, and we use the notation $C_{k\text{-part},L'}(\varrho_{L'})$ and $C_{k\text{-prod},L'}(\varrho_{L'})$, respectively.

One can also restrict the notions for (nonempty) subsystems $L' \subseteq L$ from the original system L. Let us denote the restriction of a partition $\xi \in \Pi(L)$ to subsystem L' with $\xi|_{L'} = \{X \cap L' \neq \emptyset \mid X \in \xi\} \in \Pi(L')$. It is easy to check that if $\upsilon \preceq \xi$ then $\upsilon|_{L'} \preceq \xi|_{L'}$. Let us denote the restriction of a nonempty down-set of partitions $\boldsymbol{\xi}$ to subsystem L' with $\boldsymbol{\xi}|_{L'} = \{\xi|_{L'} \mid \xi \in \boldsymbol{\xi}\}$. It is easy to check that if $\boldsymbol{\upsilon} \preceq \boldsymbol{\xi}$ then $\boldsymbol{\upsilon}|_{L'} \preceq \boldsymbol{\xi}|_{L'}$. The k-partitionability and k-producibility of the whole system L is described by the $\boldsymbol{\mu}_k$ and $\boldsymbol{\nu}_k$ down-sets of partitions in $\Pi(L)$ (see above). Then, the k-partitionability and k-producibility of the subsystem L' is described by the $\boldsymbol{\mu}_{k,L'}$ and $\boldsymbol{\nu}_{k,L'}$ down-sets of partitions in $\Pi(L')$. We have then that

$$\boldsymbol{\mu}_k|_{L'} = \boldsymbol{\mu}_{k-(|L|-|L'|),L'}, \tag{17a}$$

$$\boldsymbol{\nu}_k|_{L'} = \boldsymbol{\nu}_{k,L'}. \tag{17b}$$

(So, restricting the k-partitionability of the system makes sense only if k is larger than the number of dropped subsystems plus one; and restricting the k-producibility of the system makes sense only if k is smaller than the number of the kept subsystems.) The proofs of these are as follows. Notice that $0 \leq |\xi| - |\xi|_{L'}| \leq |L \setminus L'| = |L| - |L'|$, because the number of parts always decreases for restriction, and the largest decrease occurs when all the parts $X \in \xi$ which are not contained in L' are of size 1, then the number of empty sets, coming from $X \cap L'$, is $|L \setminus L'|$. Rearranging the relations, we have the bounds on the size of the restricted partition $|\xi| - |L \setminus L'| \leq |\xi|_{L'}| \leq |\xi|$. For the proof of (17a), we need, on the one hand, that $\forall \mu \in \boldsymbol{\mu}_k$, $\mu|_{L'} \in \boldsymbol{\mu}_{k-|L\setminus L'|,L'}$. This comes from that $k - |L \setminus L'| \leq |\mu| - |L \setminus L'| \leq |\mu|_{L'}|$, where the first inequality is $|\mu| \geq k$ (by (13a) definition of $\boldsymbol{\mu}_k$) and the second one is the above bound on the size of the restricted partition. For the proof of (17a), we need, on the other hand, that $\forall \mu' \in \boldsymbol{\mu}_{k-|L\setminus L'|,L'}$, $\exists \mu \in \boldsymbol{\mu}_k$ such that $\mu' = \mu|_{L'}$. For this, we consider the partition $\mu = \mu' \cup \mu'' \in \Pi(L)$, where $\mu'' \in \Pi(L \setminus L')$. Then $\mu|_{L'} = \mu'$ holds clearly. Let μ'' be the bottom element of $\Pi(L \setminus L')$, that is, $\mu'' := \perp_{\Pi(L\setminus L')} = \{\{i\} \mid i \in L \setminus L'\}$, then $|\mu| = |\mu'| + |\perp_{\Pi(L\setminus L')}| = |\mu'| + |L \setminus L'| \geq k$, so $\mu \in \boldsymbol{\mu}_k$. For the proof of (17b), we need, on the one

hand, $\forall \nu \in \boldsymbol{\nu}_k$, $\nu|_{L'} \in \boldsymbol{\nu}_{k,L'}$. This comes from the (13b) definition of $\boldsymbol{\nu}_k$, and from that the size of the parts of ν decreases for restriction. For the proof of (17b), we need, on the other hand, that $\forall \nu' \in \boldsymbol{\nu}_{k,L'}$, $\exists \nu \in \boldsymbol{\nu}_k$ such that $\nu' = \nu|_{L'}$. For this, we consider the partition $\nu = \nu' \cup \nu'' \in \Pi(L)$, where $\nu'' \in \Pi(L \setminus L')$. Then $\nu|_{L'} = \nu'$ holds clearly. Let ν'' be also k-producible, that is, $\nu'' \in \boldsymbol{\nu}_{k,L\setminus L'}$ then clearly $\nu \in \boldsymbol{\nu}_k$, by (13b) definition of $\boldsymbol{\nu}_k$.

Second, we would like to characterise the correlations in the whole system L, but only with respect to a coarsening, given by the partition $\upsilon = Y_1|Y_2|\dots|Y_{|\upsilon|} \in \Pi(L)$. That is, the composite subsystems $Y \in \upsilon$ become the elementary ones in the coarsened treatment. This means that, for the correlations, we use the partitions coarser than υ, the set of which is denoted by $\Pi(L,\upsilon) = \{\xi' \in \Pi(L) \mid \upsilon \preceq \xi'\} = \uparrow\{\upsilon\} \subseteq \Pi(L)$, which is a principal filter[18] in $\Pi(L)$. Then for Level I, we have the ξ'-correlation for $\xi' \in \Pi(L,\upsilon)$ with the same definition (7). For Level II, we also have the $\boldsymbol{\xi}'$-correlation for a nonempty down-set $\boldsymbol{\xi}' \subseteq \Pi(L,\upsilon)$ with the same definition (10). For the k-partitionability and k-producibility correlations, we have to denote the coarsening and we use the notation $C_{k\text{-part},\upsilon}(\varrho_L)$ and $C_{k\text{-prod},\upsilon}(\varrho_L)$, respectively. (Note that in the case of k-producibility, the minimisation in (14b) should be taken over the partitions $\nu \in \boldsymbol{\nu}_{k,\upsilon} = \boldsymbol{\nu}_k \cap \Pi(L,\upsilon) \subseteq \Pi(L,\upsilon)$ in which the parts $N \in \nu$ are the disjoint union of subsystems $Y \in \upsilon$ of number less than or equal to k.)

F. Global bounds.

The von Neumann entropy (1) takes its maximum for maximally mixed states,[2] so it is bounded by

$$S(\varrho_X) \leq \ln d_X = |X| \ln d, \tag{18}$$

where d is the (uniform) dimension of the Hilbert spaces of the elementary subsystems.

For disjoint $X, X' \in L$ subsystems, we have that the usual bipartite $X|X'$-correlation (7) is bounded by

$$C_{X|X'}(\varrho_{X\cup X'}) \leq \big(|X| + |X'|\big) \ln d. \tag{19}$$

Another bound can also be given, which is stronger, if one part is larger than the other,

$$\begin{aligned} C_{X|X'}(\varrho_{X\cup X'}) &\leq \min\big\{2\ln d_X, 2\ln d_{X'}\big\} \\ &= \min\big\{|X|, |X'|\big\} 2\ln d. \end{aligned} \tag{20}$$

The proof of this is as follows,

$$\begin{aligned} C_{X|X'}(\varrho_{X\cup X'}) &= S(\varrho_X) + S(\varrho_{X'}) - S(\varrho_{X\cup X'}) \\ &\leq S(\varrho_X) + S(\varrho_{X'}) - \big|S(\varrho_X) - S(\varrho_{X'})\big| \\ &= 2\min\big\{S(\varrho_X), S(\varrho_{X'})\big\}, \end{aligned}$$

where the (3c) Araki-Lieb triangle inequality, is used. Then using (18) completes the proof: the minimum of the entropies is bounded from above by the minimum of the upper bounds of the entropies.

Generalising the above, let us consider a partition $\xi = X_1|X_2|\dots|X_{|\xi|} \in \Pi(L)$ of the whole system L. We have then that the ξ-correlation (7) is bounded by

$$C_\xi(\varrho_L) \leq |L| \ln d. \tag{21}$$

Another bound can also be given, which is stronger, if one part is larger than the others together,

$$\begin{aligned} C_\xi(\varrho_L) &\leq \Big(\sum_{X\in\xi} 2\ln d_X - \max_{X'\in\xi}\big\{2\ln d_{X'}\big\}\Big) \\ &= \Big(|L| - \max_{X\in\xi}\big\{|X|\big\}\Big) 2\ln d. \end{aligned} \tag{22}$$

The proof of this is as follows,

$$\begin{aligned} C_\xi(\varrho_L) &= \sum_{X\in\xi} S(\varrho_X) - S(\varrho_L) \\ &\leq \sum_{X\in\xi} S(\varrho_X) - S(\varrho_{X'}) + \sum_{\substack{X\in\xi \\ X\neq X'}} S(\varrho_X) \\ &= 2\sum_{\substack{X\in\xi \\ X\neq X'}} S(\varrho_X) \qquad \text{for all } X' \in \xi, \end{aligned}$$

where the polygon inequality,

$$S(\varrho_{X'}) - \sum_{\substack{X\in\xi \\ X\neq X'}} S(\varrho_X) \leq S(\varrho_L) \qquad \text{for all } X' \in \xi,$$

is used. (This follows from the combination of the (3c) triangle inequality in the form of $S(\varrho_{X'}) - S(\varrho_{L\setminus X'}) \leq S(\varrho_L)$, holds for all $X' \subseteq L$, with the (3b) subadditivity, in the form $S(\varrho_{L\setminus X'}) \leq \sum_{X\in\xi, X\neq X'} S(\varrho_X)$.) Then using (18) completes the proof: the sum of the $|\xi| - 1$ lowest entropies is bounded from above by the sum of the $|\xi| - 1$ lowest upper-bound of the entropies. Note that (20) is a special case of this.

Having the (22) bound for the ξ-correlation (7), we can obtain the bound

$$C_{\boldsymbol{\xi}}(\varrho_L) \leq \Big(|L| - \max_{\xi\in\boldsymbol{\xi}} \max_{X\in\xi}\big\{|X|\big\}\Big) 2\ln d \tag{23}$$

for the $\boldsymbol{\xi}$-correlation (10): the minimum of ξ-correlations is bounded from above by the minimum of the upper-bounds of the ξ-correlations. This leads to the bounds for the k-partitionability correlation (14a) and the k-producibility correlation (14b)

$$C_{k\text{-part}}(\varrho_L) \leq \big(k-1\big) 2\ln d, \tag{24a}$$
$$C_{k\text{-prod}}(\varrho_L) \leq \big(|L| - k\big) 2\ln d. \tag{24b}$$

Based on the bounds (20), (22), (23) and (24), it is convenient to give all numerical results for these quantities in units of $\ln d$. On the other hand, although we do not know if the bounds (22), (23) and (24) can be

attained or not by quantum states, we emphasise that there exist stronger bounds for the correlations in classical states than for the correlations in quantum states. Indeed, applying the monotonicity of the entropy w.r.t. partial trace ($S(\varrho_{X'}) \leq S(\varrho_X)$ if $X' \subseteq X$, holds for classical states[2]), it is easy to prove, that for classical states we have half of the bounds (20), (22), (23) and (24). For example, consider the cases of two classical and two quantum dits. For classical states (embedded *locally* into quantum states), the maximally correlated one is given by $\frac{1}{d}\sum_{i=1}^d |i\rangle\langle i| \otimes |i\rangle\langle i|$, for which $C_{1|2} = \ln d$, while for quantum states, the maximally correlated one is given by $\frac{1}{d}\sum_{i,j=1}^d |i\rangle\langle j| \otimes |i\rangle\langle j|$ (projecting to the state vector $\frac{1}{\sqrt{d}}(\sum_{i=1}^d |i\rangle \otimes |i\rangle)$, for which $C_{1|2} = 2\ln d$. That is, the (20) bound is strict for quantum states (i.e., it is attained), and the (strict) bound for classical states is only the half of that. Similarly, we expect that the maximal possible values of the ξ-correlation, the $\boldsymbol{\xi}$-correlation, the k-partitionability correlation and the k-producibility correlation are strictly smaller for classical states than for quantum states.

G. Relations among the correlation measures.

We have already seen the first two relations among the correlation measures, the multipartite monotonicity of the first and second kinds in (9) and (12). We can easily have the further two,

$$C_{\boldsymbol{\xi}}(\varrho_L) \leq C_\xi(\varrho_L) \qquad \text{for all } \xi \in \boldsymbol{\xi}, \tag{25}$$

by definition (10), and

$$C_\xi(\varrho_L) \leq C_{\boldsymbol{\xi}}(\varrho_L) \qquad \text{if } \boldsymbol{\xi} \preceq \downarrow\{\xi\}, \tag{26}$$

by the multipartite monotonicity (12).

For disjoint $X, X' \in L$ subsystems, we have that the usual bipartite $X|X'$-correlation is monotonic in the sense that

$$\forall i \in X, i' \in X': \quad C_{i|i'}(\varrho_{\{i,i'\}}) \leq C_{X|X'}(\varrho_{X\cup X'}), \tag{27}$$

following from (8) and the (4) monotonicity of the relative entropy (2). (Alternatively, a different proof can be formulated exploiting the (3a) strong subadditivity of the von Neumann entropy.) From this, and the multipartite monotonicity (9), for all i, i' orbitals and for all $\xi \in \Pi(L)$ splits,

$$C_{i|i'}(\varrho_{\{i,i'\}}) \leq C_\xi(\varrho_L) \qquad \text{if } i|i' = \xi|_{\{i,i'\}}, \tag{28}$$

that is, when ξ separates i and i'. From this,

$$C_{i|i'}(\varrho_{\{i,i'\}}) \leq \min_{\xi\in\boldsymbol{\xi}} C_\xi(\varrho_L) = C_{\boldsymbol{\xi}}(\varrho_L) \quad \text{if } i|i' = \xi_*|_{\{i,i'\}}, \text{ where } \xi_* = \operatorname*{argmin}_{\xi\in\boldsymbol{\xi}} C_\xi(\varrho_L). \tag{29}$$

In particular,

$$C_{i|i'}(\varrho_{\{i,i'\}}) \leq \min_{X\subset L} C_{X|(L\setminus X)}(\varrho_L) = C_{2\text{-part}}(\varrho_L) \quad \text{if } i \in X_*, i' \in L \setminus X_*, \quad \text{where } X_* = \operatorname*{argmin}_{X\subset L} C_{X|(L\setminus X)}(\varrho_L). \tag{30}$$

Generalising the relations above, let us consider a (nonempty) subsystem $L' \subseteq L$ of the whole system L, and the $\xi|_{L'}$ restriction of a partition ξ to this subsystem. We have then

$$C_{\xi|_{L'}}(\varrho_{L'}) \leq C_\xi(\varrho_L), \tag{31}$$

following from (8) and the monotonicity of the relative entropy (2) for partial trace[4,5]. Note that the bound (28) is a special case of this. Let us consider the $\boldsymbol{\xi}|_{L'}$ restriction of a nonempty down-set of partitions $\boldsymbol{\xi}$ to this subsystem. We have then

$$C_{\boldsymbol{\xi}|_{L'}}(\varrho_{L'}) \leq C_{\boldsymbol{\xi}}(\varrho_L), \tag{32}$$

following from (31): the minimum of the $\xi|_{L'}$-correlations is bounded from above by the minimum of the larger ξ-correlations. Let us consider the k-partitionability and k-producibility of the subsystem L'. We have then

$$C_{(k\,-\,(|L|\,-\,|L'|))\text{-part},L'}(\varrho_{L'}) \leq C_{k\text{-part}}(\varrho_L), \tag{33a}$$

$$C_{k\text{-prod},L'}(\varrho_{L'}) \leq C_{k\text{-prod}}(\varrho_L), \tag{33b}$$

because of (17).

On the other hand, one can also bound level II measures of subsystems $L' \subseteq L$ by level I measures of the original system L. We have then that for a $\xi \in \Pi(L)$ split,

$$C_{\boldsymbol{\xi}'}(\varrho_{L'}) \leq C_\xi(\varrho_L) \qquad \text{if } \xi|_{L'} \in \boldsymbol{\xi}', \tag{34}$$

following from (31) and (25). In particular,

$$C_{2\text{-part},L'}(\varrho_{L'}) \leq C_\xi(\varrho_L) \quad \text{if } \xi|_{L'} \preceq X'|(L' \setminus X') \text{ for a } X' \in L', \tag{35}$$

that is, if ξ dissects L', that is, if $\xi|_{L'}$ is not the trivial split.

H. Relations for the bipartite correlation clustering.

Let us split the system into subsystems, described by the partition $\gamma = G_1|G_2|\dots|G_{|\gamma|} \in \Pi(L)$, given by the clustering based on the "connectivity" with respect to $C_{i|j}$. That is, subsystems i and j are contained in the same part $G \in \gamma$, if and only if there exists a path $i = i_1, i_2, \dots, i_p = j$ of orbitals for which $C_{i_s|i_{s+1}}(\varrho_{\{i_s,i_{s+1}\}}) \geq T_\text{b}$ for a threshold T_b for all $1 \leq s \leq p-1$. We call this *bipartite correlation clustering.*

The first point to see here is that there are nonvanishing correlations $C_{\xi'}$ and $C_{\boldsymbol{\xi}'}$ inside the parts $G \in \gamma$. Indeed, for all $\xi' \in \Pi(G)$, for all $X' \in \xi'$, there are $i' \in X'$ and $i \in G \setminus X'$ for which $C_{i|i'}(\varrho_{\{i,i'\}}) \geq T_{\mathrm{b}}$, because of the construction of γ. For these, on the other hand, $\xi'|_{\{i,i'\}} = i|i'$, so the condition of (28) holds,

$$T_{\mathrm{b}} \leq C_{i|i'}(\varrho_{\{i,i'\}}) \leq C_{\xi'}(\varrho_G). \tag{36}$$

Because this holds for all ξ', it holds also for $\xi'_* = \operatorname{argmin}_{\xi' \in \boldsymbol{\xi}'} C_{\xi'}$, so the condition of (29) holds,

$$T_{\mathrm{b}} \leq C_{i|i'}(\varrho_{\{i,i'\}}) \leq C_{\boldsymbol{\xi}'}(\varrho_G). \tag{37}$$

In particular,

$$T_{\mathrm{b}} \leq C_{i|i'}(\varrho_{\{i,i'\}}) \leq C_{2\text{-part},G}(\varrho_G). \tag{38}$$

Note that, because of (22) and (23), the bounds (36) and (37) seem to be rather weak. However, because of (24a), the bound (38) seems to be strong, depending on T_{b}.

The second point to see here is that C_γ is not necessarily weak. If there is a subsystem $L' \subseteq L$ which is dissected by γ, that is, for which $\gamma|_{L'}$ is nontrivial, and the correlation $C_{\boldsymbol{\xi}'}$ inside L' is strong (we can interpret this as the occurrence of *hidden correlations*), then C_γ is also strong,

$$C_{\boldsymbol{\xi}'}(\varrho_{L'}) \leq C_\gamma(\varrho_L) \qquad \text{if } \gamma|_{L'} \in \boldsymbol{\xi}', \tag{39}$$

which is (34). In particular,

$$C_{2\text{-part},L'}(\varrho_{L'}) \leq C_\gamma(\varrho_L) \tag{40}$$

in all cases, because all nontrivial splits are contained in $\boldsymbol{\mu}_{2,L'}$.

The third point to see here is that if C_ξ is weak for a split $\xi \in \Pi(L)$, then $\gamma \preceq \xi$, or, contrapositively, if $\gamma \npreceq \xi$ then C_ξ is strong. This comes as follows. If $\gamma \npreceq \xi$ then there is a $G \in \gamma$ which is dissected by ξ, that is, $\xi' := \xi|_G$ is not trivial. Then for all $X' \in \xi'$, there are $i' \in X'$ and $i \in G \setminus X'$ for which $C_{i|i'}(\varrho_{\{i,i'\}}) \geq T_{\mathrm{b}}$, because of the construction of γ. For these, on the other hand, $\xi'|_{\{i,i'\}} = i|i'$, so the condition of (28) holds, and using also (31), we have $T_{\mathrm{b}} \leq C_{i|i'}(\varrho_{\{i,i'\}}) \leq C_{\xi'}(\varrho_G) \leq C_\xi(\varrho_L)$.

The fourth point to see here is that one can exclude the hidden correlations among the parts of $\xi \in \Pi(L)$ if C_ξ is weak. We have just seen that if C_ξ is weak then $\gamma \preceq \xi$. If C_ξ is weak then the 2-partitionability $C_{2\text{-part},L'}(\varrho_{L'})$ is weak in every subsystem $L' \subset L$ which is dissected by ξ, that is, for which $\xi|_{L'}$ is nontrivial, that is,

$$C_{2\text{-part},L'}(\varrho_{L'}) \leq C_\xi(\varrho_L), \tag{41}$$

in the same way as (40), and here the right hand side is weak. However, if the system is large enough, then there can be several small local contributions to the global C_ξ, making it too large, even if there are no hidden correlations.

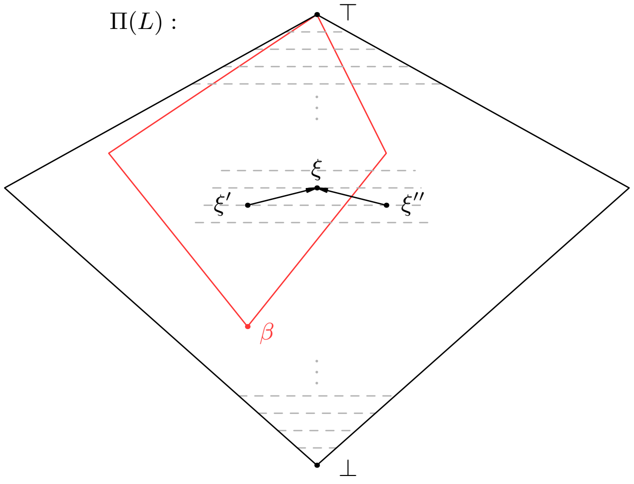

Supplementary Figure S2. Illustration for the (43) definition of multipartite correlation clustering. The up-set $\uparrow\{\beta\}$ is drawn by red line, $C_{\xi'} - C_\xi \leq T_{\mathrm{m}}$, because $\beta \preceq \xi'$, while $C_{\xi''} - C_\xi > T_{\mathrm{m}}$, because $\beta \npreceq \xi''$. (Schematic view of the partition lattice $\Pi(L)$: the arrows denote the covering relation (6) in the same way as in Fig. S1, and the dashed grey lines represent the gradation of the lattice.)

In summary, an intrinsic problem of the bipartite correlation clustering is that it is based on bipartite correlations, which are local (that is, consider only density matrices of two elementary subsystems), and which ranges in $0 \leq C_{i|j} \leq 2 \ln d$. Because of this, it is unable to grasp the multipartite correlations in a satisfactory way, unless some additivity results for $C_{i|j}$ can be proven, which does not seem to be the case.

I. Multipartite correlation clustering

Here we formulate and solve the task of dividing the whole system into weakly correlated subsystems consisting of strongly correlated elementary subsystems. We call this *multipartite correlation clustering.* That is, to obtain $\beta = B_1|B_2|\ldots|B_{|\beta|} \in \Pi(L)$, *if exists,* for which

(i) the subsystems described by the parts $B \in \beta$ are weakly correlated with one another,
(ii) the elementary subsystems $i \in B$ inside a part $B \in \beta$ are strongly correlated with one another.

It makes this notion complicated that strong and weak are ill-defined, and depend on the context. That is, although some rules of thumb might exist, we cannot formulate general thresholds for $C_{k\text{-part},B}$, $C_{k\text{-prod},B}$ and C_β independently of the situation. Instead of that, we use a different point of view, leading to a *local* strategy. For this, we have to be able to decide about a given ξ, if it is a good ansatz, or it is worth considering a ξ', which is "a bit" finer than ξ.

The first we need is to calculate the "derivative" of C_ξ with respect to ξ, that is, the difference of C_ξ for covering

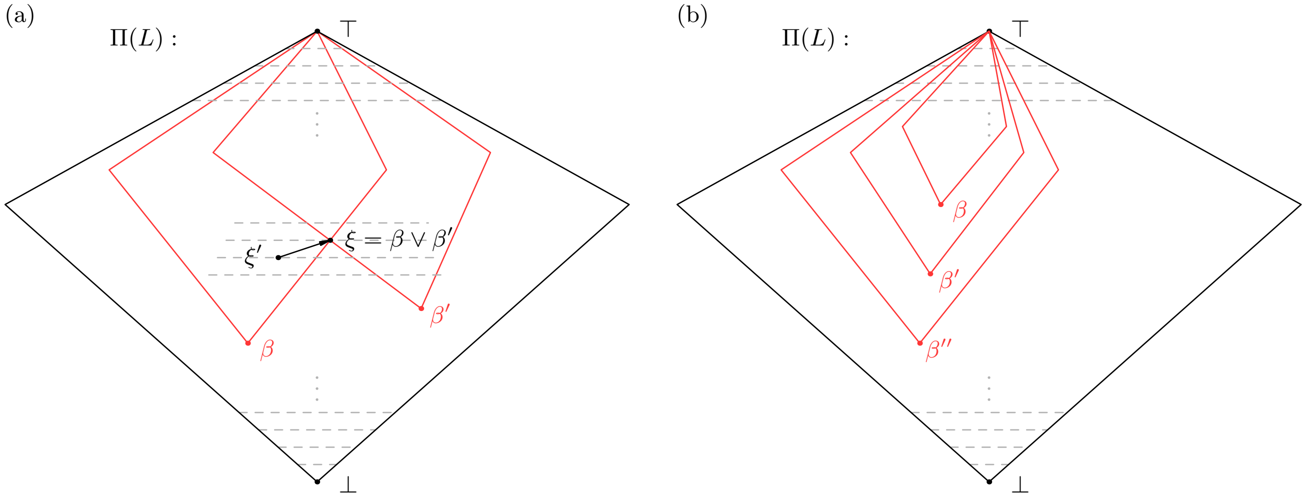

Supplementary Figure S3. Illustrations for the multipartite correlation clusterings. (a) There are no contradictory multipartite correlation clusterings. (b) There may be different compatible multipartite correlation clusterings.

ξ values. Let $\xi' \prec\!\!\cdot\, \xi$, then we have

$$C_{\xi'}(\varrho_L) - C_\xi(\varrho_L) = C_{\xi'\setminus\xi}(\varrho_X), \tag{42}$$

where X is the unique element in $\xi \setminus \xi'$, see (6). Indeed, if $\xi' \prec\!\!\cdot\, \xi$, then they have the same parts, the entropies of which cancel each other, apart that there is a unique $X_* \in \xi$, which is dissected into the disjoint $X'_{*1}, X'_{*2} \in \xi'$ parts, from which $C_{\xi'}(\varrho_L) - C_\xi(\varrho_L) = S(\varrho_{X'_{*1}}) + S(\varrho_{X'_{*2}}) - S(\varrho_{X_*}) = C_{X'_{*1}|X'_{*2}, X_*}(\varrho_{X_*})$. (Note that the right hand side in (42) is nonnegative, so C_ξ decreases with respect to the covering, which is the special case of the multipartite monotonicity (9), valid for arbitrary coarsening.)

Now we reformulate the multipartite correlation clustering (i)-(ii) as seeking β for which

$$\begin{aligned}&\text{there exists a threshold } T_\mathrm{m} > 0, \text{ such that}\\ &\forall \xi, \xi' \in \Pi(L) \text{ such that } \xi' \prec\!\!\cdot\, \xi, \text{ and } \beta \preceq \xi, \text{ then}\\ &\qquad \beta \preceq \xi' \quad \Leftrightarrow \quad C_{\xi'}(\varrho_L) - C_\xi(\varrho_L) \leq T_\mathrm{m}.\end{aligned} \tag{43}$$

(For illustration, see Fig. S2.) This means that, on the one hand, the change of the function C_ξ with ξ is small while ξ is coarser than β (ξ does not leave the up-set $\uparrow\{\beta\}$), that is, we divide only parts weakly correlated with one another (42). This is how (i) is grasped. On the other hand, the function C_ξ jumps when ξ gets not coarser than β (ξ does leave the up-set $\uparrow\{\beta\}$), that is, if we divide parts strongly correlated with one another (42). This is how (ii) is grasped. Note that, for a more robust definition, one can impose a threshold interval instead of a simple threshold value. Note also that the minimal change in C_ξ is related to the 2-partitionability of the parts in ξ,

$$\min_{\xi' \prec\!\!\cdot\, \xi}\big(C_{\xi'}(\varrho_L) - C_\xi(\varrho_L)\big) = \min_{X\in\xi} C_{\text{2-part},X}(\varrho_X), \tag{44}$$

because of (42).

There might not exist meaningful multipartite correlation clustering for a given quantum state ϱ_L, that is, there might exist no β satisfying (43). However, the notion of the multipartite correlation clustering (accordingly, the definition (43)) is strong enough to exclude the existence of more than one *contradictory* βs (that is, which are not related by coarsening). Indeed, let us take, contrapositively, that we have β and β', with possibly different thresholds $T_\mathrm{m} \leq T'_\mathrm{m}$, for which $\beta \npreceq \beta'$ and $\beta \nsucceq \beta'$. (For illustration, see Fig. S3(a).) Now let us have $\xi = \beta \vee \beta'$, the least upper bound of β and β', then for the next step $\xi' \prec\!\!\cdot\, \xi$ we have that either $\beta \not\prec \xi'$ or $\beta' \not\prec \xi'$ (it leaves either $\uparrow\{\beta\}$ or $\uparrow\{\beta'\}$). Let us choose a step $\beta' \not\prec \xi'$ and $\beta \prec \xi'$ (leaving $\uparrow\{\beta'\}$ but staying in $\uparrow\{\beta\}$), then from the definition (43) we have that $T'_\mathrm{m} < C_{\xi'}(\varrho_L) - C_\xi(\varrho_L) \leq T_\mathrm{m}$, contradicting to $T_\mathrm{m} \leq T'_\mathrm{m}$. On the other hand, more than one *compatible* β (with different thresholds T_m) might still exist (that is, which are related by coarsening). Then they form a chain, $\beta \succ \beta' \succ \beta'' \succ \dots$ ($\uparrow\{\beta\} \subset \uparrow\{\beta'\} \subset \uparrow\{\beta''\} \subset \dots$), and $T_\mathrm{m} < T'_\mathrm{m} < T''_\mathrm{m} < \dots$, this means that there are different meaningful levels of the multipartite correlation clustering, that is, different strength-scales of correlations. (For illustration, see Fig. S3(b).)

How to find β satisfying (43)? The threshold T_m in (43) seems also to be ill-defined, unless we calculate and compare C_ξ for all $\xi \in \Pi(L)$, which is infeasible even for not too large systems. ($|\Pi(L)|$ grows rapidly[38] with $|L|$.) Fortunately, we do not have to do so. It is enough to start with the trivial element $\xi = \top = \{L\}$ (with $C_\top = 0$), then carrying out *successive refinement* (that is, climbing down $\Pi(L)$ such that in each step we move from ξ to ξ' which is covered by ξ), while keeping track of the change of C_ξ. If in one step $C_{\xi'} - C_\xi$ is much larger than before, then we have to find another $\xi' \prec\!\!\cdot\, \xi$, for which $C_{\xi'} - C_\xi$ is small. If there is no such ξ', then we have reached β, that is, $\beta = \xi$. This is because in

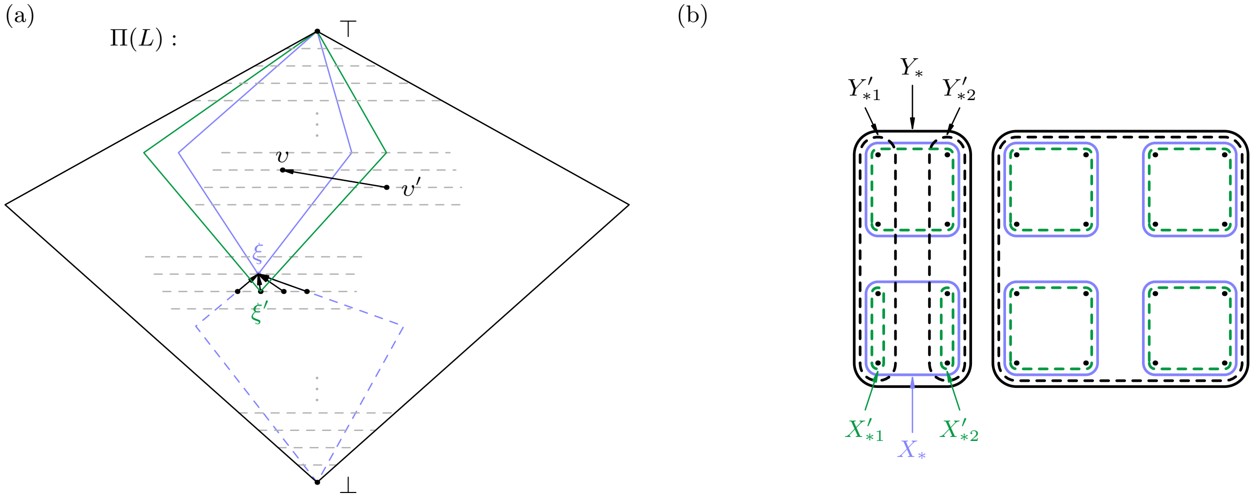

Supplementary Figure S4. Illustration for the proof of (45) for multipartite correlation clustering. (a) The up-set $\uparrow\{\xi\}$ and down-set $\downarrow\{\xi\}$ are drawn by solid and dashed blue lines. υ' leaves $\uparrow\{\xi\}$; ξ', constructed in the text, is covered by ξ, its up-set, drawn by solid green line, may or may not contain υ'. (b) The construction of ξ', see in the text: υ and υ' are drawn by solid and dashed black lines. ξ and ξ' are drawn by solid blue and dashed green lines.

general,

$$\begin{aligned}&\forall\upsilon,\upsilon'\in\Pi(L)\text{ such that }\upsilon'\prec\upsilon,\text{ and}\\&\forall\xi\in\Pi(L)\text{ such that }\xi\preceq\upsilon\text{ but }\xi\npreceq\upsilon',\text{ then}\\&\qquad\min_{\xi'\prec\xi}C_{\xi'}(\varrho_L)-C_\xi(\varrho_L)\le C_{\upsilon'}(\varrho_L)-C_\upsilon(\varrho_L).\end{aligned}\tag{45}$$

(For illustration, see Fig. S4(a).) The proof is as follows. For the right hand side, since $\upsilon'\prec\upsilon$, we have the unique $Y_*\in\upsilon$ and $Y'_{*1},Y'_{*2}\in\upsilon'$, such that $\upsilon'\setminus\upsilon=\{Y'_{*1},Y'_{*2}\}$ and $\upsilon\setminus\upsilon'=\{Y_*\}$, see (6), by which $C_{\upsilon'}(\varrho_L)-C_\upsilon(\varrho_L)=C_{\{Y'_{*1},Y'_{*2}\},Y_*}(\varrho_{Y_*})$, see (42). For the left hand side, since $\xi\preceq\upsilon$, we have that for all $X\in\xi$ there exists a $Y\in\upsilon$ such that $X\subseteq Y$, see (5). The partition υ' dissects some (at least one) parts of ξ (since $\xi\npreceq\upsilon'$). Let $X_*\in\xi$ be such a part. Note that $X_*\subseteq Y_*$, since $\xi\preceq\upsilon$, and Y_* is the one dissected by υ'. Now choose a ξ' such that $X_*\in\xi$ is also dissected by ξ' into parts "in the same way as υ'", that is, $\xi':=\big(\xi\setminus\{X_*\}\big)\cup\{X'_{*1},X'_{*2}\}$ where $X'_{*1}:=X_*\cap Y'_{*1}\subseteq Y'_{*1}$ and $X'_{*2}:=X_*\cap Y'_{*2}\subseteq Y'_{*2}$. (For illustration, see Fig. S4(b).) It is clear that $\xi'\prec\xi$, see (6). Now we have that $C_{\xi'}(\varrho_L)-C_\xi(\varrho_L)=C_{\{X'_{*1},X'_{*2}\},X_*}(\varrho_{X_*})$, see (42). Since $\{X'_{*1},X'_{*2}\}=\{Y'_{*1},Y'_{*2}\}|_{Y_*}$, the proof is completed by (31). The meaning of (45) is exactly what we need: if at a ξ the change of the correlation C_ξ is large for all the possible steps then the change of the correlation is also large if one leaves the up-set $\uparrow\{\xi\}$. So if, during the successive refinement, we follow a path in which the change of the correlation C_ξ is small, and we reach a ξ of small enough C_ξ, after which in every possible step this change becomes large, then we have reached β.

So in this way we have managed to give meaning to $C_{\xi'}-C_\xi$ being small or large, by comparing the values of C_ξ through a path from $\top$ to $\bot$. But there is a question remained: how to do the successive refinement? If, for example, we choose a wrong step *in the beginning,* with $C_{\xi'}-C_{\xi=\top}=C_{\xi'}$ being large (this is the case when $\beta\npreceq\xi'$, we leave the up-set $\uparrow\{\beta\}$), we do not notice this, and we miss the whole structure. (Choosing a wrong step later can be recognised, since the difference $C_{\xi'}-C_\xi$ becomes large, compared to the differences in the previous steps.) We can avoid this mistake if in each step we choose the step in which C_ξ changes the smallest. However, always finding the step with minimal change in C_ξ is still infeasible ($|\{\xi'\in\Pi(L)|\xi'\prec\xi\}|=\sum_{X\in\xi}(2^{|X|-1}-1)$ is still large in the beginning of the procedure). Fortunately, the bipartite correlation clustering γ, given in the previous section, often gives us a good hint. We can immediately have that the parts $G\in\gamma$ should not be dissected: the 2-partitionability $C_{2\text{-part},G}$ in G is strong (38), which determines the change of C_ξ, that is,

$$T_\mathrm{b}\le C_{\xi'}-C_\xi,\qquad\text{if }\gamma\prec\xi\text{ and }\gamma\not\prec\xi',\tag{46}$$

see (38) and (42). This reduces the possibilities for the steps in the successive refining, since it must be contained in $\uparrow\{\gamma\}$ until it reaches γ. However, in the presence of hidden correlations, that is, strong multipartite correlations among the parts of γ, we have that the change of C_ξ is high even if no part of γ is dissected (44).

J. Example for hidden correlations

Here we construct an example family of states showing hidden correlations. The smallest quantum system in which hidden correlations can occur is the system of three qubits. A general three-qubit state $\varrho_{\{1,2,3\}}$ can be

expressed in the basis of the Pauli matrices

$$\sigma_0 = \begin{bmatrix} 1 & 0 \\ 0 & 1 \end{bmatrix}, \ \sigma_1 = \begin{bmatrix} 0 & 1 \\ 1 & 0 \end{bmatrix}, \ \sigma_2 = \begin{bmatrix} 0 & -i \\ i & 0 \end{bmatrix}, \ \sigma_3 = \begin{bmatrix} 1 & 0 \\ 0 & -1 \end{bmatrix}, \tag{47}$$

with the coefficients $\varrho_{\{1,2,3\}}^{a,b,c} \in \mathbb{R}$ as

$$\varrho_{\{1,2,3\}} = \frac{1}{8} \sum_{a,b,c=0}^{3} \varrho_{\{1,2,3\}}^{a,b,c} \sigma_a \otimes \sigma_b \otimes \sigma_c. \tag{48}$$

The normalisation $\operatorname{tr} \varrho_{\{1,2,3\}} = 1$ leads to $\varrho_{\{1,2,3\}}^{0,0,0} = 1$, on the other hand, for the sake of simplicity, let us use only the σ_0 and σ_3 components, that is, $\varrho_{\{1,2,3\}}^{a,b,c} = 0$ if any of a, b, c is 1 or 2. Let then $\varrho_{\{1,2,3\}}^{3,0,0} = x$, $\varrho_{\{1,2,3\}}^{0,3,0} = y$, $\varrho_{\{1,2,3\}}^{0,0,3} = z$, $\varrho_{\{1,2,3\}}^{0,3,3} = yz$, $\varrho_{\{1,2,3\}}^{3,0,3} = xz$, $\varrho_{\{1,2,3\}}^{3,3,0} = xy$, and $\varrho_{\{1,2,3\}}^{3,3,3} = v$. For certain ranges of the parameters $(x, y, z, v) \in \mathbb{R}^4$, the resulting matrix is positive, that is, represents a state. Then, using the notation $\varrho_{\{i\}} = \operatorname{tr}_{\{j,k\}} \varrho_{\{i,j,k\}}$, $\varrho_{\{i,j\}} = \operatorname{tr}_{\{k\}} \varrho_{\{i,j,k\}}$ for all distinct $i, j, k \in \{1, 2, 3\}$, one can easily check that

$$\varrho_{\{i,j\}} = \varrho_{\{i\}} \otimes \varrho_{\{j\}}, \tag{49a}$$

while

$$\varrho_{\{1,2,3\}} = \varrho_{\{i\}} \otimes \varrho_{\{j,k\}} \quad \Longleftrightarrow \quad v = xyz. \tag{49b}$$

So, if $v \neq xyz$, then $\varrho_{\{1,2,3\}}$ is correlated with respect to any nontrivial split, although its bipartite subsystems are uncorrelated. Using the correlation measures (7) and (14), this leads to

$$C_{i|j}(\varrho_{\{i,j\}}) = 0, \tag{50a}$$

$$C_{i|j,k}(\varrho_{\{1,2,3\}}) > 0, \tag{50b}$$

$$C_{\text{2-part}}(\varrho_{\{1,2,3\}}) = C_{\text{2-prod}}(\varrho_{\{1,2,3\}}) > 0, \tag{50c}$$

$$C_{1|2|3}(\varrho_{\{1,2,3\}}) = \\ = C_{\text{3-part}}(\varrho_{\{1,2,3\}}) = C_{\text{1-prod}}(\varrho_{\{1,2,3\}}) > 0. \tag{50d}$$

Important to note that ϱ_{123} is a diagonal matrix, the diagonal elements of which can be considered as the entries of a classical three-bit state. That is, the phenomenon of hidden multipartite correlations is not a quantum feature, it exists also in states of classical systems.

II. RESULTS EMPLOYING THE MINIMAL BASIS

Here we present the results of the same calculations as in the main text, but now using STO-3G basis set.

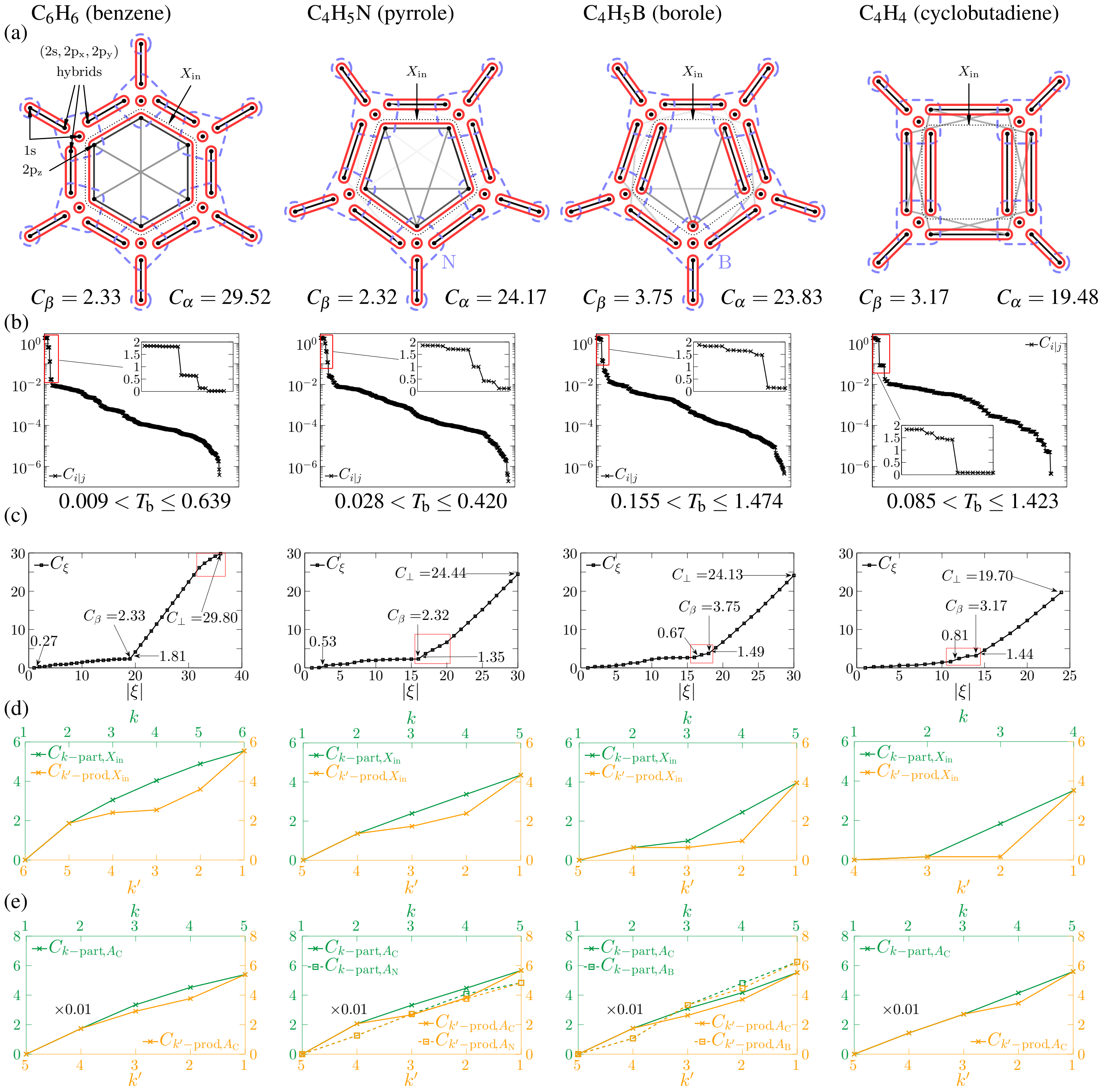

Supplementary Figure S5. Partitioning and multipartite correlations for the benzene, pyrrole, borole and cyclobutadiene molecules. The same types of data are shown as in Fig. 1 in the main text.

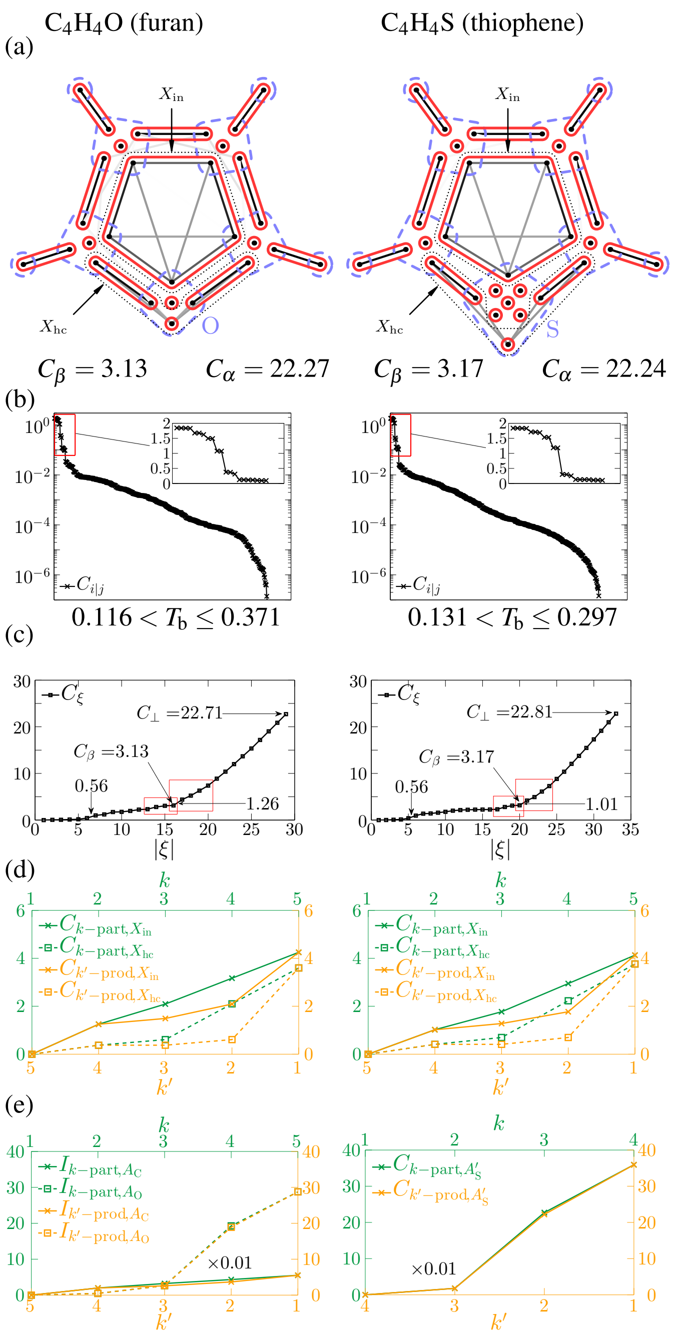

Supplementary Figure S6. Partitioning and multipartite correlations for the furan and thiophene molecules. The same types of data are shown as in Fig. 2 in the main text.

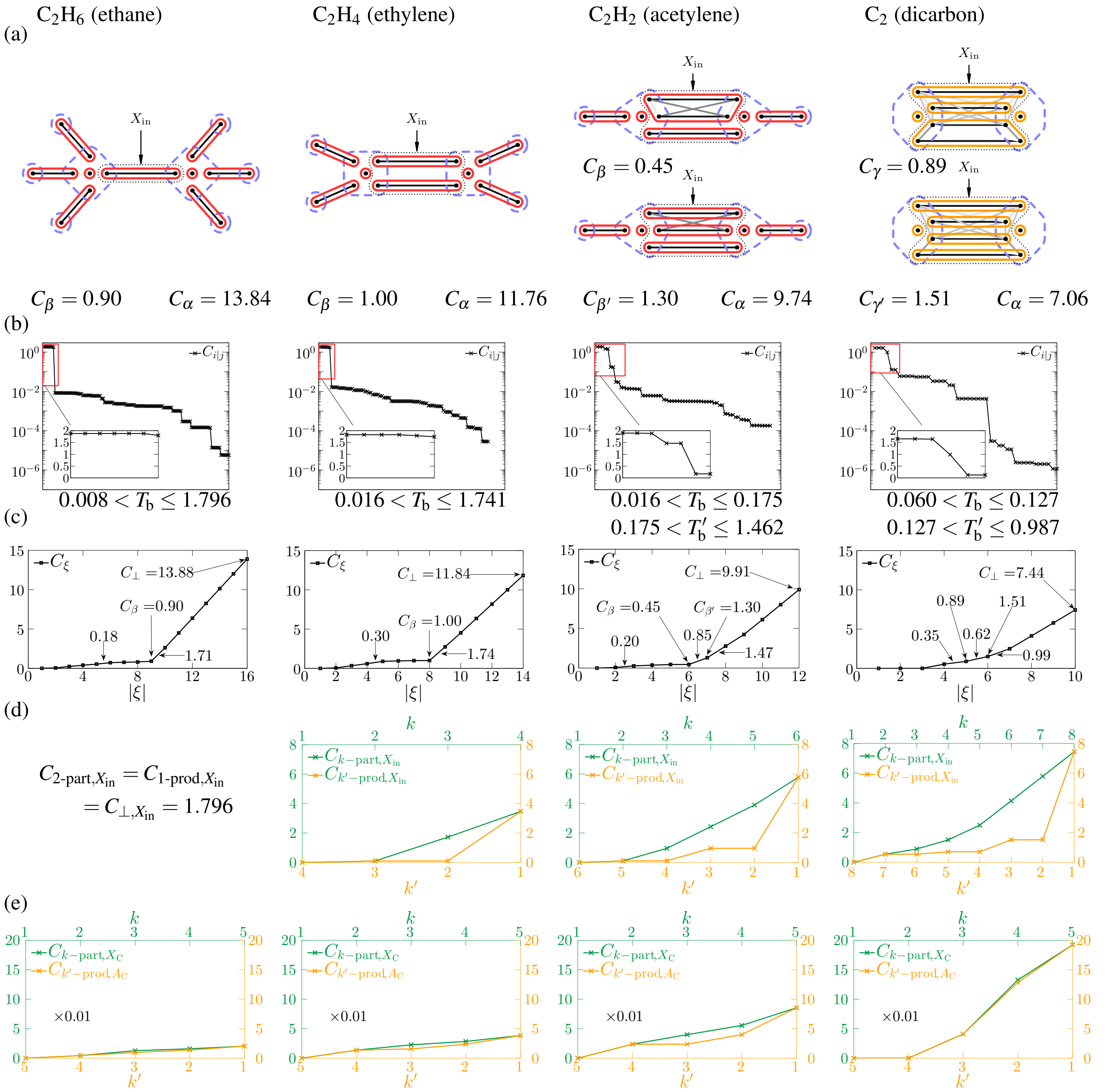

Supplementary Figure S7. Partitioning and multipartite correlations for the C_2H_{2x} molecules. The same types of data are shown as in Fig. 3 in the main text.

[1] Sz. Szalay, "Multipartite entanglement measures," *Phys. Rev. A*, vol. 92, p. 042329, Oct 2015.

[2] M. Ohya and D. Petz, *Quantum Entropy and Its Use.* Springer Verlag, 1 ed., October 1993.

[3] M. A. Nielsen and I. L. Chuang, *Quantum Computation and Quantum Information.* Cambridge University Press, 1 ed., October 2000.

[4] M. M. Wilde, *Quantum Information Theory.* Cambridge University Press, 2013.

[5] D. Petz, *Quantum Information Theory and Quantum Statistics.* Springer, 2008.

[6] H. Araki and H. Moriya, "Equilibrium statistical mechanics of fermion lattice systems," *Reviews in Mathematical Physics*, vol. 15, no. 02, pp. 93–198, 2003.

[7] H. Moriya, "Validity and failure of some entropy inequalities for car systems," *Journal of Mathematical Physics*, vol. 46, no. 3, p. 033508, 2005.

[8] M.-C. Bañuls, J. I. Cirac, and M. M. Wolf, "Entanglement in fermionic systems," *Phys. Rev. A*, vol. 76, p. 022311, Aug 2007.

[9] J. v. Neumann, "Thermodynamik quantenmechanischer gesamtheiten," *Nachrichten von der Gesellschaft der Wissenschaften zu Göttingen, Mathematisch-Physikalische Klasse*, vol. 1927, pp. 273–291, 1927.

[10] F. Hiai and D. Petz, "The proper formula for relative entropy and its asymptotics in quantum probability," *Communications in Mathematical Physics*, vol. 143, no. 1, pp. 99–114, 1991.

[11] H. Umegaki, "Conditional expectation in an operator algebra. iv. entropy and information," *Kodai Math. Sem. Rep.*, vol. 14, no. 2, pp. 59–85, 1962.

[12] T. Sagawa, "Second law-like inequalities with quantum relative entropy: An introduction," in *Lectures on Quantum Computing, Thermodynamics and Statistical Physics* (M. Nakahara and S. Tanaka, eds.), vol. 8 of *Kinki University Series on Quantum Computing*, 2012.

[13] E. H. Lieb and M. B. Ruskai, "Proof of the strong subadditivity of quantummechanical entropy," *Journal of Mathematical Physics*, vol. 14, no. 12, pp. 1938–1941, 1973.

[14] H. Araki and E. H. Lieb, "Entropy inequalities," *Communications in Mathematical Physics*, vol. 18, no. 2, pp. 160–170, 1970.

[15] D. Petz, "Quasi-entropies for finite quantum systems," *Reports on Mathematical Physics*, vol. 23, no. 1, pp. 57 – 65, 1986.

[16] D. Petz, "Monotonicity of quantum relative entropy revisited," *Reviews in Mathematical Physics*, vol. 15, no. 01, pp. 79–91, 2003.

[17] H. Kosaki, "Relative entropy of states: a variational expression," *Journal of Operator Theory*, vol. 16, no. 2, pp. 335–348, 1986.

[18] B. A. Davey and H. A. Priestley, *Introduction to Lattices and Order.* Cambridge University Press, second ed., 2002.

[19] F. Herbut, "On mutual information in multipartite quantum states and equality in strong subadditivity of entropy," *Journal of Physics A: Mathematical and General*, vol. 37, no. 10, p. 3535, 2004.

[20] D. Yang, K. Horodecki, M. Horodecki, P. Horodecki, J. Oppenheim, and W. Song, "Squashed entanglement for multipartite states and entanglement measures based on the mixed convex roof," *Information Theory, IEEE Transactions on*, vol. 55, pp. 3375–3387, July 2009.

[21] Ö. Legeza and J. Sólyom, "Quantum data compression, quantum information generation, and the density-matrix renormalization-group method," *Phys. Rev. B*, vol. 70, p. 205118, Nov 2004.

[22] Ö. Legeza, F. Gebhard, and J. Rissler, "Entanglement production by independent quantum channels," *Phys. Rev. B*, vol. 74, p. 195112, Nov 2006.

[23] G. Lindblad, "Entropy, information and quantum measurements," *Communications in Mathematical Physics*, vol. 33, pp. 305–322, 1973.

[24] R. Horodecki, "Informationally coherent quantum systems," *Physics Letters A*, vol. 187, no. 2, pp. 145 – 150, 1994.

[25] K. Modi, T. Paterek, W. Son, V. Vedral, and M. Williamson, "Unified view of quantum and classical correlations," *Phys. Rev. Lett.*, vol. 104, p. 080501, Feb 2010.

[26] R. Horodecki, P. Horodecki, M. Horodecki, and K. Horodecki, "Quantum entanglement," *Rev. Mod. Phys.*, vol. 81, pp. 865–942, Jun 2009.

[27] Sz. Szalay, "Quantum entanglement in finite-dimensional hilbert spaces," *arXiv [quant-ph]*, p. 1302.4654, 2013.

[28] L. Amico, R. Fazio, A. Osterloh, and V. Vedral, "Entanglement in many-body systems," *Rev. Mod. Phys.*, vol. 80, pp. 517–576, May 2008.

[29] C. H. Bennett, H. J. Bernstein, S. Popescu, and B. Schumacher, "Concentrating partial entanglement by local operations," *Phys. Rev. A*, vol. 53, pp. 2046–2052, Apr 1996.

[30] E. Schmidt, "Zur theorie der linearen und nichtlinearen integralgleichungen. i. teil: Entwicklung willkürlicher funktionen nach systemen vorgeschriebener," *Math. Ann.*, vol. 63, pp. 433–476, 1907.

[31] Sz. Szalay, "Separability criteria for mixed three-qubit states," *Phys. Rev. A*, vol. 83, p. 062337, Jun 2011.

[32] Sz. Szalay and Z. Kökényesi, "Partial separability revisited: Necessary and sufficient criteria," *Phys. Rev. A*, vol. 86, p. 032341, Sep 2012.

[33] A. Acín, D. Bruß, M. Lewenstein, and A. Sanpera, "Classification of mixed three-qubit states," *Phys. Rev. Lett.*, vol. 87, p. 040401, Jul 2001.

[34] O. Gühne, G. Tóth, and H. J. Briegel, "Multipartite entanglement in spin chains," *New Journal of Physics*, vol. 7, no. 1, p. 229, 2005.

[35] M. Seevinck and J. Uffink, "Partial separability and entanglement criteria for multiqubit quantum states," *Phys. Rev. A*, vol. 78, p. 032101, Sep 2008.

[36] M. Seevinck and J. Uffink, "Sufficient conditions for three-particle entanglement and their tests in recent experiments," *Phys. Rev. A*, vol. 65, p. 012107, Dec 2001.

[37] G. Tóth and O. Gühne, "Separability criteria and entanglement witnesses for symmetric quantum states," *Applied Physics B*, vol. 98, no. 4, pp. 617–622, 2010.

[38] "The On-Line Encyclopedia of Integer Sequences, A000110." Bell or exponential numbers: ways of placing n labeled balls into n indistinguishable boxes.
